# Translation of Overlapping Open Reading Frames Promoted by Type 2 IRESs in Avian Calicivirus Genomes

**DOI:** 10.3390/v16091413

**Published:** 2024-09-04

**Authors:** Yani Arhab, Tatyana V. Pestova, Christopher U. T. Hellen

**Affiliations:** Department of Cell Biology, SUNY Downstate Health Sciences University, Brooklyn, NY 11203, USA; yani.arhab@downstate.edu (Y.A.); tatyana.pestova@downstate.edu (T.V.P.)

**Keywords:** calicivirus, IRES, translation initiation, overlapping reading frame, Ebp1

## Abstract

Caliciviruses have positive-sense RNA genomes, typically with short 5′-untranslated regions (5′UTRs) that precede the long open reading frame 1 (ORF1). Exceptionally, some avian caliciviruses have long 5′UTRs containing a picornavirus-like internal ribosomal entry site (IRES), which was likely acquired by horizontal gene transfer. Here, we identified numerous additional avian calicivirus genomes with IRESs, predominantly type 2, and determined that many of these genomes contain a ~200–300 codon-long ORF (designated ORF1*) that overlaps the 5′-terminal region of ORF1. The activity of representative type 2 IRESs from grey teal calicivirus (GTCV) and *Caliciviridae* sp. isolate yc-13 (RaCV1) was confirmed by in vitro translation. Toeprinting showed that in cell-free extracts and in vitro reconstituted reactions, ribosomal initiation complexes assembled on the ORF1* initiation codon and at one or two AUG codons in ORF1 at the 3′-border and/or downstream of the IRES. Initiation at all three sites required eIF4A and eIF4G, which bound to a conserved region of the IRES; initiation on the ORF1* and principal ORF1 initiation codons involved eIF1/eIF1A-dependent scanning from the IRES’s 3′-border. Initiation on these IRESs was enhanced by the IRES *trans*-acting factors (ITAFs) Ebp1/ITAF_45_, which bound to the apical subdomain Id of the IRES, and PTB (GTCV) or PCBP2 (RaCV1).

## 1. Introduction

Viruses depend on the cellular translation apparatus. They have evolved strategies to usurp it for the synthesis of viral proteins, to exploit its capabilities to maximize utilization of the coding capacity of viral mRNAs, and to suppress the translation of cellular mRNAs during infection, particularly of mRNAs that are induced as part of the innate immune response [1,2].

The *Caliciviridae* family comprises at least eleven genera of viruses and has a broad host range, including birds, fish, mammals and reptiles [3]. Caliciviruses have single-stranded, positive-sense RNA genomes of 6.4–8.5 kb with a VPg (viral protein genome-linked) covalently bound to the 5′-terminus, a short 5′-UTR and a 3′-terminal poly(A) tail [4]. A subgenomic mRNA that is 3′-coterminal to the genomic RNA is synthesized during infection, and it is also VPg-linked. Calicivirus genomes generally contain two or three open reading frames (ORFs): the 5′-proximal ORF1 encodes a large polyprotein that is cleaved by a virus-encoded protease to yield non-structural proteins. The structural proteins VP1 and VP2 are encoded by ORF2 and ORF3 and are expressed from the subgenomic mRNA. These ORFs overlap, and RNA elements upstream of the ORF2 stop codon subvert post-termination recycling and promote reinitiation on the ORF3 initiation codon [5,6,7]. Exceptionally, murine norovirus subgenomic mRNAs encode an overlapping ORF4 within the VP1 coding region [8,9].

The canonical initiation process [10] begins with binding of the eukaryotic initiation factor (eIF)2-GTP/Met-tRNA_i_^Met^ complex, eIF1, eIF1A and eIF3 to the ribosomal 40S subunit to form a 43S preinitiation complex. Cellular mRNAs have a 5′-terminal m^7^GpppG cap that is recognized by the eIF4E cap-binding subunit of eIF4F, which also contains eIF4A (helicase) and eIF4G subunits. It recruits the 43S complex to the capped end of the mRNA. The 43S complex scans downstream to locate the initiation codon in a process that requires eIF1 and eIF1A and that involves the unwinding of mRNA by eIF4F, which is aided by eIF4B. Scanning on highly structured 5′-UTRs also requires the DExH-box protein DHX29. The establishment of base pairing between the initiation codon and the anticodon of Met-tRNA_i_^Met^ arrests the scanning 43S complex and forms a 48S complex after the eIF5-induced hydrolysis of eIF2-bound GTP. The subsequent eIF5B-mediated reorientation of Met-tRNA_i_^Met^, joining of a 60S ribosomal subunit and release of factors forms an 80S ribosome that is competent to begin translation.

The 5′-ends of calicivirus mRNAs are linked to VPg rather than to a m^7^Gppp cap, so the initiation of translation is independent of the cap-eIF4E interaction. Instead, VPg recruits eIF4F and associated factors such as eIF3 via interactions with eIF4E and eIF4G and exploits the stress granule assembly factor G3BP1 to promote the binding of the 40S subunit to the VPg-linked mRNA [11,12,13]. This distinct mechanism of ribosomal recruitment to calicivirus mRNAs may account for their preferential translation in infected cells in which the translation of cellular mRNAs is impaired [14]. Subsequent steps in this initiation process remain uncharacterized. Caliciviruses therefore utilize strategies such as the synthesis of subgenomic mRNA, synthesis of a polyprotein that is proteolytically cleaved to yield mature products, translation of linked ORFs and overlapping ORFs to maximize utilization of their genomic coding capacity, and the non-canonical initiation and termination–reinitiation mechanisms for the translation of viral mRNA.

The 5′UTRs of a subset of calicivirus genomes contain an IRES and can therefore exploit alternative non-canonical mechanisms for the initiation of translation. IRESs are complex, highly structured RNA elements that form structural classes, each of which uses a different subset of initiation factors to mediate initiation by a distinct mechanism (Table 1) [15]. Type 1 and type 2 IRESs, exemplified by poliovirus and encephalomyocarditis virus (EMCV), respectively, are ~450 nt long and consist of five domains, designated II–VI in type 1 and H–L in type 2 IRESs. Both types of IRES contain highly conserved, functionally important motifs. In type 2 IRESs, they include a C-rich loop, the GNRA tetraloop and an AAA motif in apical arms of domain I [16,17,18,19], an A-rich stem-loop wedged between J and K domains [20], and an apical bipartite motif in domain J [21,22,23]. Type 1 and type 2 IRESs both have a 3′-terminal Yn-Xm-AUG motif, in which a Yn pyrimidine tract (*n* = 8–10 nt) is separated by a spacer (m = 18–20 nt) from an AUG triplet [24]. This triplet is the initiation codon for the viral polyprotein in type 2 IRESs, although initiation can also occur downstream of it [25,26,27]. Type 5 IRESs are also ~450 nt long and are chimeric, containing a Yn-Xm-AUG motif and constituent elements that resemble domains from type 1 and type 2 IRESs [28,29].

Initiation on type 1 and type 2 IRESs relies on their specific interaction with the central eIF4A-binding domain of eIF4G [20,21,22,23,30,31,32,33]. These IRESs can consequently function without eIF4E and the N-terminal region of eIF4G to which it binds, which is commonly cleaved from eIF4G by virus-encoded proteases during infection, shutting off cellular translation. 48S complex formation on type 2 IRESs requires eIF2, eIF3, the central domain of eIF4G and eIF4A, and is enhanced by eIF4B [30,32,33]; scanning to AUG codons downstream of the Yn-Xm-AUG motif additionally requires eIF1 and eIF1A [27]. These IRESs also commonly require specific IRES trans-acting factors (ITAFs). The principal ITAF for type 1 IRESs is the poly(C) binding protein 2 [31], whereas type 2 IRESs require the pyrimidine tract binding protein (PTB) and in some instances, ErbB3-binding protein 1 (Ebp1, also known as ITAF_45_) [30,32,33,34,35,36].

Type 4 IRESs, exemplified by hepatitis C virus (HCV), are ~330nt long and employ a fundamentally different mechanism. They bind directly to the 40S subunit, positioning the initiation codon in the ribosomal P site so that the recruitment of eIF2-GTP/Met-tRNA_i_^Met^ forms a 48S complex without the involvement of group 4 eIFs [37,38]. Subunit joining on IRES types 1, 2, 4 and 5 is mediated by eIF5 and eIF5B.

Type 6 IRESs, exemplified by the Cricket paralysis virus intergenic region, are the smallest IRESs, and they function by the most streamlined mechanism, binding directly to 40S subunits and/or 80S ribosomes to bypass the entire initiation process and instead commencing directly with elongation [39,40].

**Figure 1:**
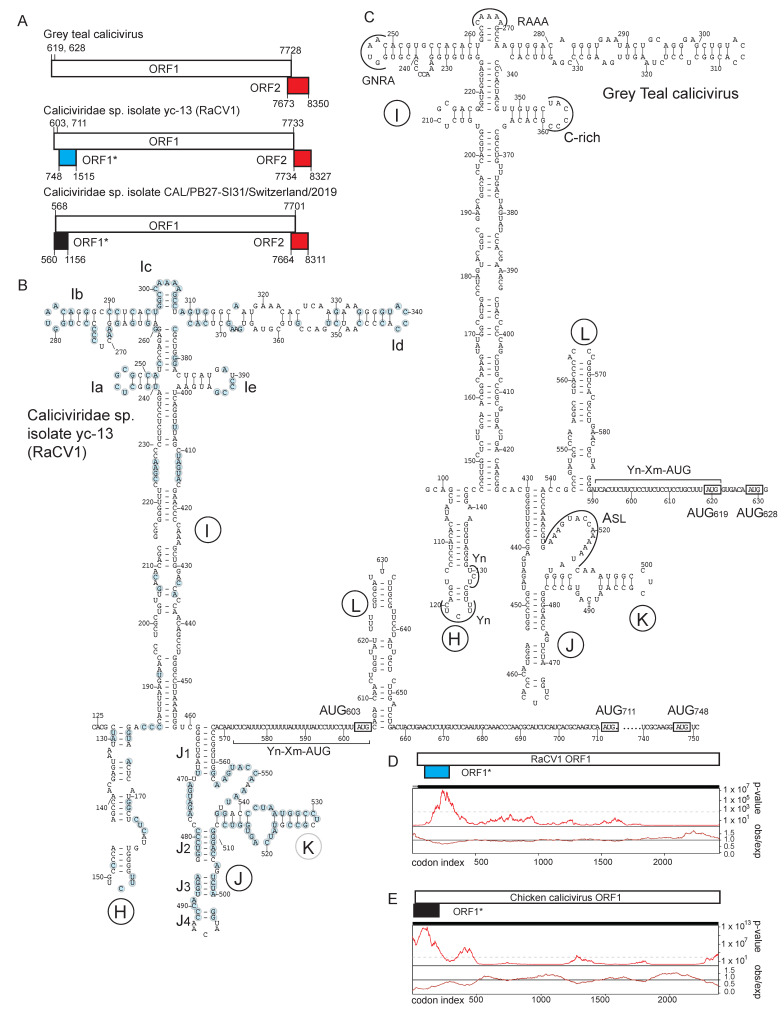
Models of the genomes and type 2 IRESs of representative avian caliciviruses. (**A**) Schematic representation of the coding regions of grey teal calicivirus (GTCV) isolate MW09, *Caliciviridae* sp. isolate yc-13 (RaCV1) and *Caliciviridae* sp. Isolate CAL/PB27-SI31/Switzerland/2019. ORF1 is cleaved into mature non-structural proteins, ORF2 encodes the VP2 capsid protein. ORF1* is the overlapping open reading frame identified in this report. (**B**,**C**) Models of the secondary structures of (b) RaCV1 and (c) GTCV IRESs. IRES domains are labeled sequentially from H to L; nucleotides that are identical in 10 of the 13 sequences in Supplementary Figure S1 are indicated by blue circles. (**B**) Subdomains in domain I are labeled sequentially from Ia to Ie, and helices in domain J are labeled J_1_ to J_4_. (**C**) The model is annotated to show conserved sequence motifs, including oligopyrimidine (Yn) motifs in domain H, GNRA, RAAA and C-rich motifs at the apex of domain I, the ASL domain in the J-K domain and the Yn-Xm-AUG motif downstream of the J-K domain. Nucleotides are numbered, and the initiation codons AUG_603_, AUG_711_ and AUG_748_ (RaCV1) and AUG_619_ and AUG_628_ (GTCV) are boxed. (**D**,**E**) Synonymous-site variability in a subset of avian caliciviruses. (**D**) Above: map of RaCV1 ORF1 and the overlapping ORF1*. Below: analysis of synonymous-site variability in an alignment of eight avian calicivirus sequences. (**E**) Above: map of Chicken calicivirus isolate Chicken/NLD/2019/V_M_030_calici_8 ORF1 and the overlapping ORF1*. Below: analysis of synonymous-site variability in an alignment of eleven chicken calicivirus sequences. (**D**,**E**) The lower panel (obs/exp) (darker red lines) indicates the relative amount of synonymous-site variability as represented by the ratio of the observed number of synonymous substitutions to the expected number, (**D**) in a 101-codon and (**E**) in a 75-codon sliding window. The upper panels (brighter red lines) show the corresponding *p*-values. (**A**,**D**,**E**) ORF2 coding regions are colored red, and the two classes of ORF1* are colored blue and black, respectively.

Seven caliciviruses belonging to the *Nacovirus* genus of *Caliciviridae* and to another currently unclassified clade of caliciviruses were found by us to contain elements that are closely related to picornavirus type 2 IRESs (e.g., Figure 1B,C), one to have a type 4 IRES and one to have a type 5 IRES [41]. A subsequent report identified a type 2 IRES in Trumpeter swan calicivirus [42]. Some members of these two genera (e.g., Goose calicivirus strain N [43] and Turkey calicivirus isolate L11043 [44]) have short 5′ non-coding regions, whereas Duck calicivirus 2 strain DuCV-2_B6 [45] has a 275 nt long 5′UTR but lacks an IRES. It is not known whether these sequences correspond to incomplete genomes or to genomic mRNAs that naturally have short 5′UTRs. This point is addressed below.

We previously determined that the type 4 IRES of Ruddy turnstone calicivirus mediates initiation by a mechanism that involves the direct binding of the IRES to the 40S subunit [41] analogous to that used by type 4 IRESs such as that of HCV, completely bypassing the requirement for the eIF4 group of initiation factors. In the present report, we determined that several additional calicivirus genomes contain IRESs, identified conserved ORFs (designated ORF1*) that overlap ORF1 in distinct groups of caliciviruses, and characterized the mechanism of initiation of translation of ORF1 and ORF1* mediated by these type 2 IRESs. These studies add to the understanding of viral genome compaction strategies, of viral translation mechanisms and of the dissemination of genetic elements between viruses by recombination.

## 2. Materials and Methods

### 2.1. Sequences

Sequences were analyzed from the following caliciviruses (name followed by Genbank accession number): Avocet calicivirus isolate MW21 (MH453804.1), Bird calicivirus isolate Cormcali01 (OR062614.1), *Caliciviridae* sp. isolate 361bR-k141_109560 (MZ679042.1), *Caliciviridae* sp. isolate hwf182cal1 (MT138020.1), *Caliciviridae* sp. isolate xftoti59cal1 (MT138028.1), Calicivirus chicken/V0021/Bayern/2004 (NC_075411.1), Chicken calicivirus ChCV-052 (MH992118.1), Chicken calicivirus isolate GA/1478/2003 (MN810874.1), Chicken calicivirus isolate GA/1479/2004 (MN810875.1), Chicken calicivirus isolate CaliciD62/2013 (KM254170.1), Chicken calicivirus strain RS/BR/15/1R-1 (MG846434.1), Chicken calicivirus strain RS/BR/15/3S-1 (MG846429.1), Chicken calicivirus strain RS/BR/15/4S-1 (MG846430.1), Chicken calicivirus strain RS/BR/15/6R (MG846433.1), Chicken calicivirus strain RS/BR/15/1R-1 (MG846434.1), Chicken calicivirus strain RS/BR/2015 (KY120883.1), Duck calicivirus 2 strain DuCV-2_B6 (MN175552.1), Duck calicivirus 2 strain DuCV-2_B76 (MN175556.1), Duck calicivirus isolate MW20 (MH453811.1), Goose calicivirus strain N (KJ473715.1), Goose calicivirus isolate MA/CHN/2018 (MN068022.1), Goose calicivirus isolate H146 (KY399947.1), Grey teal calicivirus (MK204392.1), Duck calicivirus isolate MW20 (MH453811.1), Mute swan feces associated calicivirus strain Abbotsbury/A/2016 (MW588067.1), Norovirus G1 (NC_001959.2), Pink-eared duck calicivirus I (MK204416.1), *Caliciviridae* sp. isolate yc-13 red-crowned crane virus (KY312552.1), Ruddy Turnstone calicivirus A isolate MW19 (MH453861.1), Temminck’s stint calicivirus isolate SIB_91 (ON815296.1), Trumpeter swan calicivirus (OP271827.1), Turkey calicivirus isolate L11043 (JQ347522.1), Wilkes virus isolate Antarctic11 (MT025075.1) from the following caliciviruses (name followed by GenBase accession number): Goose calicivirus isolate NC-19BV-37-k77-711556 (C_AA057594.1) and Duck calicivirus isolate NC-19BV-45-k77-747816 (C_AA057597.1), and from the following calicivirus *metagenome*-assembled genomes (MAGs) (name followed by Genbank accession number): Bavaria virus isolate A5_2S5_SA_2022 (PP228889.1), *Bavovirus* sp. isolate Chicken/NLD/2019/V_M_032_calici_1 (MW684839.1), *Bavovirus* sp. isolate Chicken/NLD/2019/V_M_021_calici_5 (MW684836.1), *Bavovirus* sp. isolate Chicken/NLD/2019/V_M_029_calici_1 (MW684837.1), Chicken calicivirus isolate Environment/NLD/2019/VE_8_calici_66 (MW684834.1), Chicken calicivirus isolate Chicken/NLD/2019/V_M_019_calici_3 (MW684835.1), Chicken calicivirus isolate Chicken/NLD/2019/V_M_030_calici_8 (MW684838.1), Chicken calicivirus isolate Chicken/NLD/2019/V_M_036_calici_2 (MW684840.1), Chicken calicivirus isolate Chicken/NLD/2019/V_M_041_calici_11 (MW684841.1), Chicken calicivirus isolate Chicken/NLD/2019/V_M_050_calici_2 (MW684842.1), Chicken calicivirus isolate Chicken/NLD/2019/V_M_051_calici_5 (MW684843.1), Chicken calicivirus isolate Chicken/NLD/2019/V_M_052_calici_7 (MW684844.1), Chicken calicivirus isolate Chicken/NLD/2019/V_M_053_calici_1 (MW684845.1), Chicken calicivirus isolate Chicken/NLD/2019/V_M_056_calici_1 (MW684847.1) *Caliciviridae* sp. isolate CAL/PB30-SII34/Switzerland/2019 (OM469263.1), *Caliciviridae* sp. isolate CAL/PB10-HII34X/Switzerland/2019 (OM469260.1), Caliciviridae sp. isolate CAL/PB27-SI31/Switzerland/2019 (OM469262.1), *Caliciviridae* sp. isolate bbr034cal1 (MT138025.1), *Caliciviridae* sp. isolate ybb044cal01nc (MT138022.1), *Caliciviridae* sp. isolate mag166cal1 (MT138021.1), *Caliciviridae* sp. isolate cftwhg06cal1 (MT138017.1), *Caliciviridae* sp. isolate fmg067cal1nc (MT138023.1), *Caliciviridae* sp. isolate zftwig05sapo1nc (MT138019.1), Duck calicivirus isolate PI202116/k141_160805 (PP512781.1), Shelduck calicivirus isolate MW17 (MH453874.1).

Sequences were analyzed from the following picornaviruses (name followed by Genbank accession number): Anativirus A (AV; formerly Duck picornavirus TW90A) (AY563023.1), Mute swan feces associated sapelovirus 1 strain Abbotsbury/B/2016 (MW588168.1) and Red-necked stint Picornavirus B-like (MK204387.1).

### 2.2. Nucleotide Sequence Alignment and Modelling of RNA Structures

Candidate IRES sequences were identified in the NCBI database (accessed 1 June 2024) using BLASTN (http://www.ncbi.nlm.nih.gov/BLAST/) to search nucleotide collection (nr/nt) sequences, using the following parameters: E, 1000; word size, 11; match/mismatch scores, 1/1; gap costs, 2/1. Hits were characterized by six-frame translation (https://www.ncbi.nlm.nih.gov/orffinder/ and http://molbiol.ru/eng/scripts/01_13.html) to identify potential initiation codons and open reading frames.

Sequence alignments of IRESs made using Clustal Omega (http://www.ebi.ac.uk/Tools/msa/clustalo/) were adjusted manually using established IRES structures as guides [41,46]. Pairwise comparisons were made using EMBOSS matcher (https://www.ebi.ac.uk/jdispatcher/psa/emboss_matcher) with default parameters. Structural elements were modeled using Mfold (http://unafold.rna.albany.edu/?q=mfold) [47] with default parameters. ORF1* sequences were aligned using Clustal Omega with default parameters.

Synonymous site conservation was analyzed with SYNPLOT2 [48], using codon-respecting alignments of (a) complete ORF1 sequences from *Caliciviridae* sp. isolate yc-13 (RaCV1), Duck calicivirus 2 strain DuCV-2_B6, Goose calicivirus isolate NC-19BV-37-k77-711556, Goose calicivirus strain N, Pink-eared duck calicivirus I, Turkey calicivirus isolate L11043, Wilkes virus isolate Antarctic11 and Caliciviridae sp. isolate xftoti59cal1 and a 101 codon sliding window and (b) ORF1* and ORF1 sequences from calicivirus *metagenome*-assembled genomes (MAGs) with Genbank accession numbers: MW684838.1, MW684840.1, MW684841.1, MW684842.1, MW684843.1, MW684844.1, MW684845.1, MW684847.1, OM469263.1, OM469260.1 and OM469262.1 and a 75 codon sliding window.

### 2.3. Phylogenetic Analysis

Sequences were aligned using Clustal Omega, and gaps and ambiguously aligned regions were removed using BMGE [49], in both instances using default settings, and maximum- likelihood phylogenetic trees were inferred using PhyML with Smart Model Selection (LG + G + I + F model), implemented in NGPhylogeny [50,51]. The phylogenetic tree with branch supports was visualized using iTOL [52] and annotated using Adobe Illustrator.

### 2.4. Plasmids

Expression vectors for His_6_-tagged eIF1, eIF1A [53], eIF4A, eIF4B [33], eIF4A (R362Q) [54], eIF4GI_653–1115_, eIF4GI_736–1008_ and eIF4GI_736–988_ [55], cysteine-less and T830C eIF4GI_736–1115_ [21], eIF4GI_653–1600_ [30], eIF5 [56], DHX29 [57], *Escherichia coli* methionyl tRNA synthetase [58] and nPTB [59] have been described. The vector pET15b-His_6_-Ebp1 for expression in *E. coli* of N-terminally His_6_-tagged Ebp1 was made by inserting the appropriate DNA sequence between Nde1/BamH1 sites of pET15b (GenScript, Piscataway NJ, USA). Ebp1 cysteine substitution mutants were made (Genscript) by introducing individual surface-exposed cysteine residues at positions 22, 37, 55, 65, 94, 131, 165, 208, 221, 254, 271, 311 and 360.

The transcription vectors for dicistronic DC Aichivirus IRES-containing mRNA [28] and monocistronic EMCV nt 373–1656 mRNA [41] have been described. pUC57-FMDV nt 278–891 was made by inserting FMDV O_1_K nt 278–891 (GenBank: X00871.1) flanked by SnaBI and NdeI sites and an upstream T7 promoter into the EcoRV site of pUC57 (Genscript).

pUC57[T7-Stem-GTCV] was prepared (Genscript) by synthesizing a DNA fragment with flanking HindIII and BamH1 sites that corresponded to a T7 promoter, a stable hairpin (ΔG = −32.40 kcal/mol) and GTCV nt 1–1494) with tandem UAA-UGA stop codons inserted after nt 1461 and substitutions to replace codons at positions 216, 225, 241, 254 and 263 in the ORF initiating at AUG_528_ with AUG codons to increase the methionine content of the 278 amino acid (a.a.)-long (31.7 kDa) coding sequence). This DNA fragment was inserted into pUC57. This plasmid was used by GenScript to generate [GUAA-UACG], [GUAA-CCUU], [destab. Dom L], [AUG_619_AAA], [AUG_628_AAA], [AUG_723_AAA] and [AUG_619_AUC] substitution mutants and the [ΔIb] and [ΔStem 1] deletion mutants.

The transcription vector pUC57[T7-Stem-XL-GTCV] was prepared (GenScript) by inserting a 1533 nt long BstEII-BamHI DNA fragment of pUC57[T7-Stem-GTCV] corresponding to the entire modified GTCV sequence (i.e., with tandem stop codons and AUG codon substitutions) into DC-Stem-ΔXL-HalV [60] to replace the intergenic region and ORF2 by GTCV nt 1–1494.

pUC57-T7-Stem-RaCV1[nt 1–1386] was prepared (GenScript) by inserting DNA corresponding to a T7 promotor and a stable hairpin (ΔG = −32.40 kcal/mol) linked to nt 1–1386 of the RaCV1 genome or a stable hairpin (ΔG = −33.40 kcal/mol) linked to RaCV1 nt 1–1538 between HindIII and BamHI sites of pUC57. The RaCV1 sequence was modified (Genscript) to generate [UAA_612_], [UAA_612_UAA_720_], [GAA366-8CUC],[ GUG_357–9_CUC], [ACC_343–5_UAG], [CCC_340–2_AAA], substitution mutants, [ΔC341], [Δnt 334–347] and [Δnt 317–366] deletion mutants and [Stem1 + AUG_630_], [Stem2 + AUG_630_] and [Stem3 + AUG_630_] insertion mutants.

The transcription vector pUC57-T7-Stem-RaCV1[nt 1–1538] was prepared (GenScript) by inserting DNA corresponding to a T7 promotor and a stable hairpin (ΔG = −33.40 kcal/mol) linked to RaCV1 nt 1–1538 with flanking ApaI and BamHI sites into pUC57. The RaCV1 sequence was modified to place a UAA stop codon at nt 1497–1499 and to replace codons at positions 245 and 289 in the ORF initiating at AUG_603_ (ORF1) and at positions 173, 180 and 182 in the ORF initiating at AUG_748_ (ORF1*) with AUG codons to increase the methionine content of the 298 amino acid (a.a.)-long (33.5 kDa) ORF1 and 182 a.a.-long (19.8 kDa) ORF1* coding sequences. This plasmid was used (GenScript) to generate [GUAA-UACG] and [GUAA-CCUU] substitution mutants and the [Δ1b] deletion mutant.

pUC57[T7-DC RaCV1 wt] and pUC57[T7-DC RaCV1(GUAA_280–283-_CCUU)] were prepared by inserting DNA corresponding to RaCV1 nt 124–797 flanked by 5′-XhoI and 3′-BamHI sites into DC Aichivirus [28], replacing the Aichivirus IRES by the RaCV1 IRES, and a fragment of the adjacent 287 a.a.-long (31.3 kDa) ORF. pUC57[T7-DC RaCV1 wt] was used (Genscript) to generate [AUG_603_-context], [AUG_603_-AAG], [AUG_711_AAA], [AUG_630_], [AUG_669_], [J_2_-dest] and [J_3_-dest] substitution mutants and [Stem1 + AUG_630_], [Stem2 + AUG_630_] and [Stem3 + AUG_630_] insertion mutants.

pUC57 plasmids were linearized by EcoRV. mRNAs were transcribed with T7 RNA polymerase.

### 2.5. Purification of Initiation Factors, Elongation Factors and Ribosomal Subunits

Ribosomal 40S and 60S subunits, native eIF2, eIF3, eIF5B, eEF1H, eEF2 and total aminoacyl-tRNA synthetases were purified from RRL (Green Hectares, Oregon, WI), and recombinant eIF1, eIF1A, eIF4A, eIF4A^R362Q^, eIF4B, eIF4GI_653–1115_, eIF4GI_736–988_ eIF4GI_736–1008_, cysteine-less and T830C eIF4GI_736–1115_, eIF4GI_653–1600_, nPTB, PCBP2 and *E. coli* methionyl tRNA-synthetase were expressed in *E. coli* BL21(DE3) and purified as described [31,40,61]. Recombinant Ebp1 was expressed in 4 L *E. coli* BL21 (DE3) for 18–20 h at 16 °C after induction with 1 mM IPTG and purified by Ni-NTA-agarose affinity chromatography. Ebp1 was then purified by FPLC on a monoQ 5/50 GL column with fractions being collected across a linear 100–500 mM KCl gradient: Ebp1 eluted at 200 mM KCl. Native total (Σ) calf liver tRNA (Promega, Madison WI) was aminoacylated with all amino acids using native aminoacyl-tRNA synthetases [61] or with methionine and recombinant *E. coli* methionyl-tRNA synthetase [58].

### 2.6. Assembly and Analysis of Ribosomal Complexes

The 48S complexes were assembled by incubating 50 nM RNA with 100 nM 40S subunits, 125 nM 60S, 125 nM total tRNAs, 0.5 µM eIF2, 0.2 µM eIF3, 1 µM eIF1 (unless otherwise indicated), 1 µM eIF1A, 0.8 µM eIF4A, 0.1 µM eIF4B, 0.8 µM eIF4G_653–1600_, 0.25 µM Ebp1 and 0.25 µM PCBP2 or 0.25 µM PTB for 15 min at 37 °C in 40 µL buffer A (20 mM Tris, pH 7.5, 2 mM dithiothreitol, 0.25 mM spermidine, 2.5 mM MgCl_2_) supplemented with 1 mM ATP, 0.5 mM GTP, 100 mM KCl and 1.5 U/µL RNAse inhibitor. The elongation competence of the resulting 48S complexes was assayed by incubation with 0.25 µM eIF5, 125 nM eIF5B and 120 nM 60S subunits for 10 min, which was followed by the addition of 100 nM eEF1H and 110 nM eEF2 and incubation for another 10 min. The positions of 48S and 80S complexes were determined by primer extension inhibition using AMV reverse transcriptase (Promega) and γ^32^P-end-labeled primers 5′-GAGATGCGTTGGGTTTGCAA-3′ (complementary to RaCV1 nt 678–697), 5′-GGCACATTCACGACAATTG-3′ (complementary to RaCV1 nt 788–806), 5′-ATTGTAGGCAGTGTAGGCGT-3′ (complementary to RaCV1 nt 808–827) or 5′-GAATCGACGGGTGTAACA-3′ (complementary to GTCV nt 693–710).

### 2.7. In Vitro Translation

Monocistronic mRNAs (4 pmol) or dicistronic mRNAs (2 pmol) were translated for 1 h at 37 °C using the Flexi RRL system (Promega) in 20 µL reaction mixtures with 70 mM KCl, 1.3 mM MgCl_2_, 0.4 mM complete amino acids (minus methionine) and 0.5 mCi/mL [^35^S]methionine (43.5 TBq/mmol) supplemented with ~20 pmol eIF4A^wt^ or dominant-negative eIF4A^R362Q^ as indicated. Translation products were analyzed by electrophoresis using Nu-PAGE 4–12% Bis-Tris-Gel (Invitrogen, Carlsbad, CA), which was followed by autoradiography.

### 2.8. Fe(II)-BABE Modification of eIF4G_726–1115_ and Ebp1

Ebp1 and eIF4G_726–1115_ mutant proteins were derivatized with Fe(II)-BABE (Dojindo, Rockville, MD, USA) [62,63] by incubating 30 µM eIF4G_726–1115_ or Ebp1 with 1 mM Fe(II)-BABE in 100 mL buffer B (80 mM HEPES, 260 mM KCl, 10% glycerol) for 30 min at 37 °C. Derivatized proteins were separated from unincorporated reagent by buffer exchange on Amicon 10k filter units and stored at −80 °C.

### 2.9. Directed Hydroxyl Radical Cleavage

RNA/[Fe(II)-BABE]-Ebp1 complexes were formed by incubating 2 pmol RNA and 5 pmol [Fe(II)-BABE]-Ebp1 in 50 µL buffer C (20 mM Tris, pH 7.5, 0.25 mM spermidine, 2.5 mM MgCl_2_, 0.1 mM dithiothreitol) supplemented with 1 mM ATP, 0.5 mM GTP and 1.5 U/µL RNAse inhibitor for 15 min at 37 °C. IRES/eIF4G_726–1115_ complexes with or without eIF4A were prepared as described [21]. To generate hydroxyl radicals, reaction mixtures were incubated on ice for 10 min, supplemented with 0.06% H_2_O_2_ and 5 mM ascorbic acid [62] and incubated on ice for 10 min. Reactions were quenched by adding 20 mM thiourea. RNAs were phenol-extracted, ethanol-precipitated and analyzed by toeprinting using AMV reverse transcriptase and the γ^32^P-end-labeled primers 5′-GTCTACTAAATCAGCCCGAC-3′ (complementary to RaCV1 nt 458–477), 5′-GCAATAGGAACGCAAGAATCGC-3′ (complementary to RaCV1 nt 625–646), 5′-GAGATGCGTTGGGTTTGCAA-3′ (complementary to RaCV1 nt 678–697), 5′-TTTACCGGAGCGGTATAGTC-3′ (complementary to GTCV nt 488–507), 5′-GGAGAAGAAGTGSTCCCTAG-3′ (complementary to GTCV nt 584–603), 5′-GAGTGTCGCTTGTTACC-3′ (complementary to FMDV nt 603–597) or 5′-GCAAGTCTCTTGTTCCATGG-3′ (complementary to EMCV nt 863–844), as appropriate.

### 2.10. Quantification and Statistical Analysis

All in vitro experiments were repeated at least three times, and they included technical and biological replicates. Representative gel images are shown.

## 3. Results

### 3.1. Type 2 and Type 4 IRESs in Avian Caliciviruses

Our initial analysis identified picornavirus-like type 2, type 4 and type 5 IRESs in seven caliciviruses from the *Nacovirus* genus of *Caliciviridae* and another currently unclassified clade of caliciviruses [41]. We now identified type 2 IRESs in *Caliciviridae* sp. isolate 361bR-k141_109560 [64], Goose calicivirus isolate NC-19BV-37-k77-711556 [65], Duck calicivirus isolate PI202116/k141_160805 and four closely related chicken caliciviruses [66,67] as well as partial type 2 IRESs in Duck calicivirus isolate NC-19BV-45-k77-747816 and some chicken caliciviruses (Supplementary Figure S1). Several recently described genomes contain short, possibly incomplete 5′UTRs that have high levels of nucleotide sequence identity to 3′ terminal regions of the full-length IRES-containing 5′UTRs. Pairwise sequence identity between these putative calicivirus type 2 IRESs from the 5′ border of domain H to the 3′ border of domain J is high (44–100%). They contain elements that are important for the function of picornavirus type 2 IRESs, including a C-rich loop, a GNRA tetraloop and an AAA motif in apical arms of domain I, an A-rich stem-loop that wedges between the minor grooves of J and K domains, a bipartite sequence/structural motif at the apex of domain J and a 3′-terminal Yn-Xm-AUG motif. These elements coincide with the most highly conserved nucleotides in calicivirus type 2 IRESs (Figure 1B), suggesting that these motifs are also important for their function.

Several structural properties distinguish calicivirus type 2 IRESs from the best-characterized picornavirus counterparts, such as those of EMCV and FMDV. Stemloop Id emerges from the four-way junction at the apex of domain I and is 10–15 nt long in EMCV and FMDV IRESs [46], whereas the equivalent domain in calicivirus IRESs is hyperextended (~67 nt long). The sequence downstream of the J-K domain is highly variable: some calicivirus type 2 IRESs such as that of grey teal calicivirus (GTCV) contain a hairpin (domain L) between the J-K domain and the Yn-Xm-AUG motif (Figure 1C), whereas in others, such as *Caliciviridae* sp. isolate yc-13 (“Red-crowned crane calicivirus” (RaCV1) [68], domain L overlaps the AUG triplet of the Yn-Xm-AUG motif (Figure 1B). In picornavirus type 2 IRESs, domain L is located upstream of the Yn-Xm-AUG motif [46,69], and mutational analysis of the FMDV IRES suggested that it makes a minor contribution to IRES activity [18,70]. The separation between the Yn-Xm-AUG motif and the ORF1 initiation codon ranges from 6 nt in GTCV to 216 nt in Duck calicivirus isolate MW20. The sequence divergence between calicivirus IRESs, structural differences at their 3′ borders and the variable separation between the Yn-Xm-AUG motif and ORF1 together suggest that the recombinational exchange of IRESs between caliciviruses and picornaviruses may have occurred on multiple occasions. This conclusion is supported by our analysis of other recently reported calicivirus genomes, which revealed that Temminck’s stint calicivirus [71] has a type 4 IRES that is related to that of Red-necked stint Picornavirus B-like [72] (74% nucleotide identity), and that Bird calicivirus isolate Cormcali01 from cormorants [73] contains a type 4 IRES that is related to those of the picornaviruses Anativirus A1 isolate TW90A [74,75] and Mute swan feces associated sapelovirus 1 [76] (>60% nucleotide identity).

### 3.2. An Overlapping Open Reading Frame in Avian Calicivirus Genomes

In most of these type 2 IRESs, the AUG of the Yn-Xm-AUG motif is in poor nucleotide context, suggesting that it will be bypassed and that translation of the principal open reading frame (ORF1) initiates at a downstream location. In addition to multiple potential initiation codons downstream of the motif and in frame with ORF1, many of these IRES-containing genomes contain an overlapping ORF, which is designated here as ORF1* (Figure 1A). The ORF1* amino acid sequences form two principal conserved groups, which are epitomized by RaCV1 and *Caliciviridae* sp. Isolate CAL/PB27-SI31/Switzerland/2019, respectively (Supplementary Figures S2 and S3). The former group is divergent (33–100% a.a. sequence identity and, with the exception of incomplete sequences, 213–317 amino acids (a.a.) long), but similarity is high over the C-terminal ~70 a.a.-long region, whereas the latter group is highly homologous (197–198 a.a.-long; 90–100% a.a. sequence identity). The ORF1* initiation codons in both groups almost all have suboptimal nucleotide context with pyrimidine residues at −3 and/or at +4 positions. Notably, some calicivirus genomes that lack 5′-terminal IRESs nevertheless encode ORF1* sequences. Thus, Turkey calicivirus isolate L11043, Goose calicivirus N and Duck calicivirus 2 strain DuCV-2_B6, which have 5′UTRs that are 8, 10 and 275 nt long, respectively, all encode an RaCV1-like ORF1* sequence. Only four of the fifteen chicken caliciviruses contain complete type 2 IRESs, but they all contain ORF1* sequences. The presence in caliciviruses that have short 5′UTRs of ORF1* sequences that are closely related to ORF1* sequences in IRES-containing genomes suggests that ORF1* is conserved because it has an important function. It could possibly be translated following end-dependent and IRES-mediated modes of initiation, although alternatively, ORF1*-containing genomes with short 5′UTRs may simply be incomplete. A few avian caliciviruses, such as GTCV, lack ORF1*.

**Figure 2:**
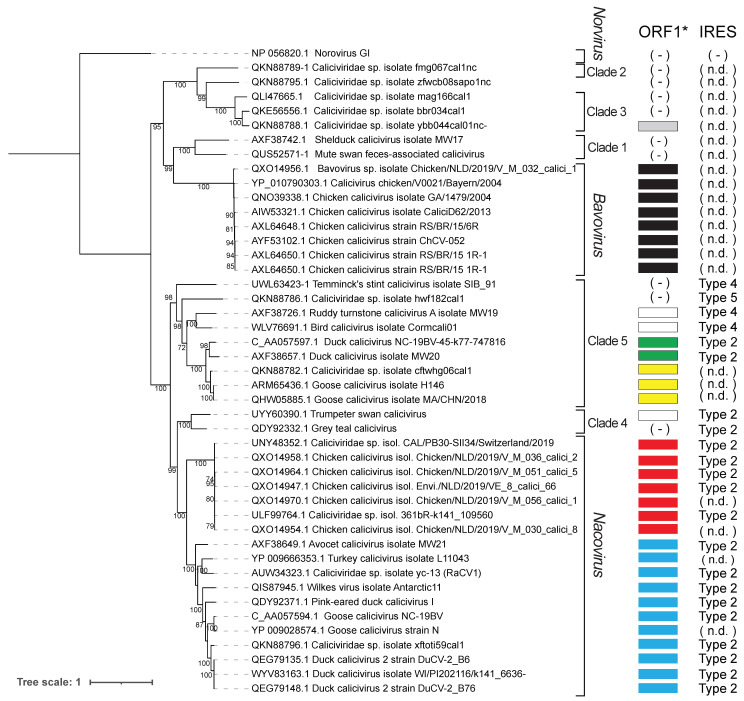
Maximum likelihood phylogenetic tree of the ORF1 polyprotein of avian members of the virus family *Caliciviridae*, which where analyzed using amino acid sequences deposited in Genbank or derived computationally from nucleotide sequence deposited in Genbase. Viruses are identified by name and Genbank protein or Genbase nucleotide accession number, as appropriate. Bootstrap values over 70% are shown. The scale bar represents the number of amino acid substitutions per site. Viruses are assigned to established genera and to clades proposed by Canuti et al. [42]. The key to the right indicates that IRES type in the viral genome, and the occurrence of overlapping ORF1* sequences, color-coded to indicate the different conserved groups (light blue: members listed in Supplementary Figure S2; red: members listed in Supplementary Figure S3; yellow, green and grey: members listed in Supplementary Figures S4A, S4B and S4C, respectively; and black: members listed in Supplementary Figure S5). White rectangles indicate potential ORF1* sequences in Ruddy turnstone calicivirus, Bird calicivirus isolate Cormacali01 and Trumpeter swan calicivirus genomes. (n.d.) indicates genomes that may be incomplete so that definitive conclusions regarding the presence of IRESs and/or ORF1* sequences cannot be made. ( - ) indicates genomes that appear to lack ORF1* or recognized IRES sequences. This analysis excluded ORF sequences that are <50 a.a long. Bavovirus 5′NTRs are long and we cannot exclude that they might have IRES activity.

Phylogenetic analysis indicates that the two major ORF1*-encoding classes of avian calicivirus genomes form coherent groups, and that the GTCV and Trumpeter swan calicivirus genomes constitute a separate cluster (Figure 2). These observations are consistent with the results of previously reported phylogenetic analyses [42,65,66,67,71,73,74,77]. Notably, numerous other calicivirus genomes also encode ORF1* sequences, forming additional groups that include (a) Duck calicivirus isolates MW20 and NC-19BV-45-k77-747816, (b) *Caliciviridae* sp. isolate cftwhg06cal1 and Goose calicivirus isolates H146 and MA/CHN/2028, (c) *Caliciviridae* sp.isolate ybb044cal01nc (Supplementary Figure S4), (d) numerous members of the *Bavovirus* genus (Supplementary Figure S5) and (e) Ruddy turnstone calicivirus A isolate MW19, Bird calicivirus isolate Cormacali01 and Trumpter swan calicivirus. The first two of these groups of ORFs show limited similarity to the major group epitomized by RaCV1, and like them, they initiate downstream of the ORF1 initiation codon. The disposition of coding sequences at the 5′-end of *Bavovirus* genomes is more complex: (i) many genomic sequences appear to be incomplete, so that open reading frame(s) commonly begin at the 5′-terminal nucleotide, encoding an N-terminal extension of the annotated ORF, and (ii) a second conserved ORF (ORF1′) either overlaps ORF1 or is in the same reading frame and is separated from it by a short intergenic region (Supplementary Figure S5). ORF1* elements therefore appear to be characteristic of most avian calicivirus genomes and to form several distinct groups.

Overlapping functional elements in mRNAs such as coding regions constrain sequence evolution and can be identified on the basis of reduced synonymous site variation [48]. Analysis of the aligned ORF1 coding sequences of (a) eleven chicken calicivirus and (b) nine other avian calicivirus genomes revealed a region of enhanced synonymous site conservation in each group that ended at the conserved location of the ORF1* stop codon, which is consistent with the presence of functionally important, translated overlapping ORFs (Figure 1D,E).

To locate ORF1 and ORF1* initiation codons, and to ascertain whether ORF1* is accessible to initiating ribosomes, we assayed the translation of mRNAs containing GTCV and RaCV1 5′UTRs in rabbit reticulocyte lysate (RRL) and analyzed initiation in reactions reconstituted in vitro from the individual purified components of the translation apparatus.

### 3.3. Initiation of Translation on the Grey Teal Calicivirus (GTCV) IRES

**Figure 3:**
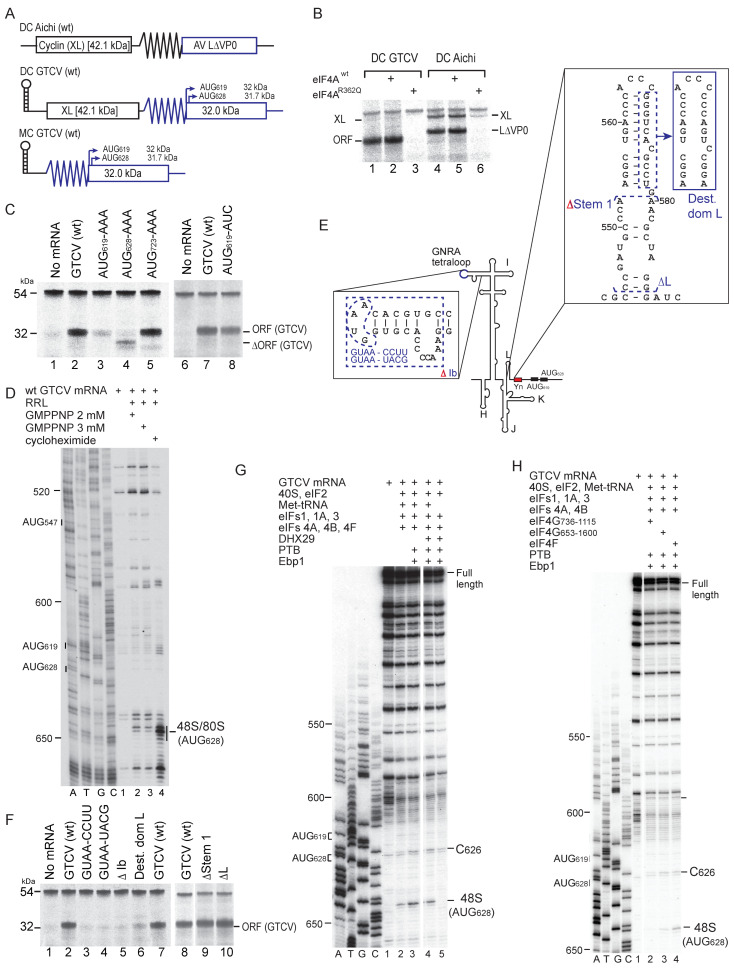
The mechanism of initiation on the grey teal calicivirus (GTCV) IRES. (**A**) Schematic representations of monocistronic (MC) GTCV and dicistronic (DC) GTCV and Aichivirus mRNAs, with IRES sequences represented by zigzag lines, a stable hairpin placed upstream of the first cistron (the cyclin B2 (XL) ORF), and potential initiation codons and corresponding translation products indicated on the downstream GTCV ORF. (**B**,**C**,**F**) Translational activity of the GTCV and Aichivirus IRESs in RRL, showing (**B**) their sensitivity to inhibition by dominant-negative eIF4A^R362Q^ and the effect (**C**) of substitution of potential GTCV initiation codons and (**F**) of substitutions and deletion mutations in subdomains Ib and domain L. The XL translation product and products resulting from initiation at GTCV and Aichivirus initiation codons resolved by SDS-PAGE are indicated. (**D**) Toeprint analysis of 48S complexes and 80S ribosomes formed on wt GTCV MC mRNA in RRL in the presence of GMPPNP and cycloheximide, as indicated. (**E**) Model of the IRES, annotated to show mutations in subdomains Ib and domain L. (**G**,**H**) Toeprinting analysis of 48S complex formation on GTCV MC mRNA in the presence of 40S subunits, unfractionated Met-tRNA_i_^Met^, eIFs, PTB1, Ebp1, DHX29, eIF4F, eIF4G_736–1115_ or eIF4G_653–1600_, as indicated. Toeprints caused by 48S complexes assembled on AUG_628_ and a factor-induced stop at C_626_ are indicated on the right. A dideoxynucleotide sequence generated with the same primer was run in parallel on each gel (lanes A, T, G, C) to show the corresponding viral sequence. Lanes in panel (**H**) separated by a white line were juxtaposed from the same gel. Lanes 6–8 of panel (**C**) and lanes 8–10 of panel (**F**) were derived from the same gel.

To test the activity of the GTCV 5′UTR as an IRES, we assayed the translation in RRL of a dicistronic mRNA with a stable 5′-terminal hairpin followed by part of the cyclin B2 (‘XL’) coding sequence, the GTCV 5′UTR and part of the adjacent ORF (Figure 3A). The GTCV ORF was robustly translated, whereas XL translation was very weak, which is consistent with the inhibitory effect of the 5′-terminal hairpin on end-dependent initiation [78] (Figure 3B). Translation of both ORFs in this mRNA and in a control dicistronic mRNA containing the Aichivirus IRES [28] was almost completely inhibited by dominant-negative eIF4A^R362Q^, which acts by sequestering eIF4G. The GTCV 5′UTR therefore contains an IRES, and initiation on it requires eIF4A and eIF4G.

The principal ORF in the GTCV genome contains in-frame AUG_523_ and AUG_547_ triplets upstream of the Yn tract and in-frame AUG_619_, AUG_628_ and AUG_723_ codons downstream of it (Figure 1C). AUG_619_ is part of the Yn-Xm-AUG motif, and it has poor nucleotide context (UUUAUGG), like the AUG codons in the Yn-Xm-AUG motif of other type 2 IRESs, which are consequently weak initiation codons and are frequently bypassed by ribosomes during initiation [25,27,79,80]. The in vitro translation of monocistronic GTCV mRNA with a 5′-terminal hairpin (ΔG = −32.4 kcal/mol) to block end-mediated ribosomal attachment yielded a single 32 kDa product, which is consistent with initiation at AUG_619_ or AUG_628_ (Figure 3C, lane 2). Translation was strongly impaired by AUG_619_AAA substitutions, whereas AUG_628_AAA substitutions eliminated translation of the 32 kDa product but yielded low levels of a 28 kDa product, which is consistent with initiation at the next downstream AUG triplet, AUG_723_. Notably, an AUG_619_AUC substitution reduced but did not abrogate translation of the 32 kDa product (Figure 3C, lane 8), whereas AUG_723_AAA substitutions had no effect on its synthesis (Figure 3C, lane 5). These results are consistent with a model in which AUG_619_ is part of the Yn-Xm-AUG motif which acts to promote internal ribosomal entry, so that mutation of it reduces initiation at all IRES-dependent sites, whereas AUG_628_ is the start codon for the polyprotein, and its substitution leads to initiation downstream. The identity of the initiation codon was confirmed by toeprinting ribosomal complexes formed on the IRES in RRL in the presence of GMP-PNP (which arrests initiation at the stage of 48S complex formation), and by toeprinting 80S ribosomes formed on the IRES in the presence of cycloheximide (which inhibits elongation) (Figure 3D). Ribosomes and ribosomal initiation complexes arrest primer extension ^+^15–^+^17 nt from the ^+^1 nucleotide of the codon in the P site, yielding characteristic toeprints. The appearance of toeprints ^+^15–^+^17 from AUG_628_ confirmed that this triplet is the primary initiation codon for GTCV ORF1.

The GTCV IRES contains a GNRA tetraloop at the apex of subdomain Ib (Figure 1E) that is characteristic of picornavirus type 2 IRESs and is important for their function. Consistently, CCUU and UACG substitutions in the tetraloop and deletion of the apical half of subdomain Ib all abrogated IRES function (Figure 3F, lanes 3–5). Whereas the tetraloop is a conserved feature of all type 2 IRESs, domain L is variable in terms of sequence, structure, and position relative to the JK domain and the Yn-Xm-AUG motif [46,81,82]. The effect of deleting part or all of domain L (Figure 3E; Figure 3F, lanes 9, 10) indicated that it does not have an essential function. However, destabilization of the apical half of this domain almost abolished IRES activity (Figure 3F, lane 6), which is possibly due to the disruption of the optimal spatial orientation of elements such as the JK domain and the Yn-Xm-AUG motif.

In vitro reconstitution showed that 40S subunits, Met-tRNA^Met^_i_, eIFs 1, 1A, 2, 3, 4A, 4B and 4F were sufficient for 48S complex formation on the GTCV and confirmed AUG_628_ as the initiation codon (Figure 3G, lane 2). Initiation required initiator tRNA (Figure 3G, lane 5) and was enhanced by PTB and Ebp1 (Figure 3G, lanes 2, 3); eIF4F could be substituted by eIF4A and eIF4G_653–1600_ and less effectively by eIF4A and eIF4G_736–1115_ (Figure 3H, lanes 2–4). DHX29 enhances initiation on type 5 IRESs but not on type 2 (EMCV), type 4 or type 6 IRESs [28,29], and consistently, it did not enhance initiation on the GTCV IRES at AUG_628_ (Figure 3G, lanes 3, 4). A reverse transcriptase (RT) stop appeared at C_626_ independently of 48S complex formation, PTB and Ebp1 (Figure 3G, lanes 2–5; Figure 3H, lanes 2–4), indicating that it is likely induced by canonical initiation factors, potentially eIF4G/eIF4A, which induces conformational changes in the EMCV IRES that lead to the appearance of RT stops downstream of the Yn-Xm-AUG motif [21].

### 3.4. Initiation of Translation on the Red-Capped Crane Calicivirus 1 (RaCV1) IRES

**Figure 4:**
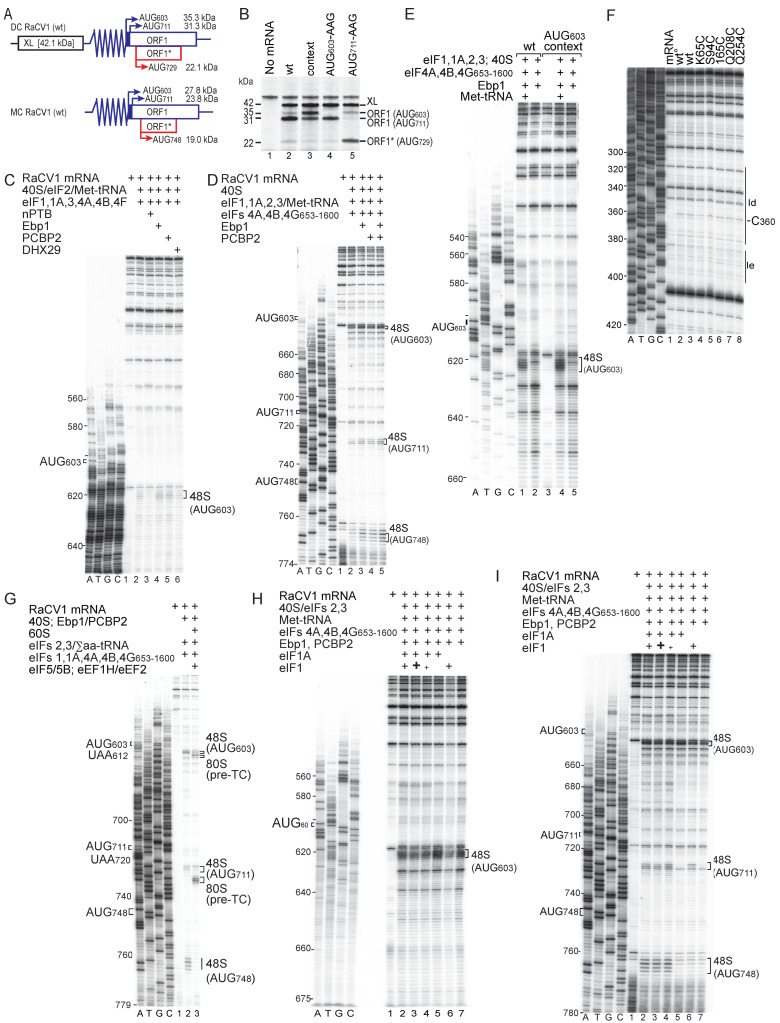
Initiation factor requirements for initiation on the RaCV1 IRES**.** (**A**) Schematic representation of monocistronic (MC) and dicistronic (DC) RaCV1 mRNAs showing IRESs represented by zigzag lines, the upstream cyclin B2 (XL) ORF and the downstream RaCV1 ORF1 and ORF1* with potential initiation codons and corresponding translation products. (**B**) Translation activity of the RaCV1 IRES and initiation codon selection in RRL mediated by the wt RaCV1 IRES and variants with improved nucleotide context for AUG_603_ or substitution of AUG_603_ or AUG_711_ by AAG codons. The XL translation product and products resulting from initiation at AUG_603_ or AUG_711_ in ORF1 and at AUG_729_ upstream of and in-frame with ORF1* resolved by SDS-PAGE are indicated. (**C**–**E**,**G**–**I**) Toeprint analysis of formation of 48S initiation complexes and 80S pre-termination complexes (pre-TC) on MC RaCV1 mRNA in the presence of combinations of 40S subunits, 60S subunits, Met-tRNA_i_^Met^ or total aminoacylated tRNAs (Σaa-tRNAs), eIFs, Ebp1, PCBP2, DHX29, eEF1H and eEF2, as indicated. In panels (**H**,**I**), lanes 2, 3 and 4 included 1 µM eIF1, 3 µM eIF1 and 0.3 µM eIF1, respectively. Toeprints caused by 48S complexes on AUG_603_, AUG_711_ and AUG_748_, and by pre-TCs assembled on UAA_612_ and UAA_720_ are indicated on the right. A dideoxynucleotide sequence generated with the same primer was run in parallel on each gel (lanes A, T, G, C) to show the corresponding viral sequence. (**F**) Primer extension analysis conducted using the wt RaCV1 IRES and unmodified wt Ebp1 (wt°; lane 2) or Fe(II)BABE-modified wt or mutant Ebp1 with substitutions as indicated (lanes 3–8). The cDNA product labeled C_360_ terminated at this nucleotide. Reference lanes A, T, G, C show the wt RaCV1 sequence generated with the same primer.

**Figure 5:**
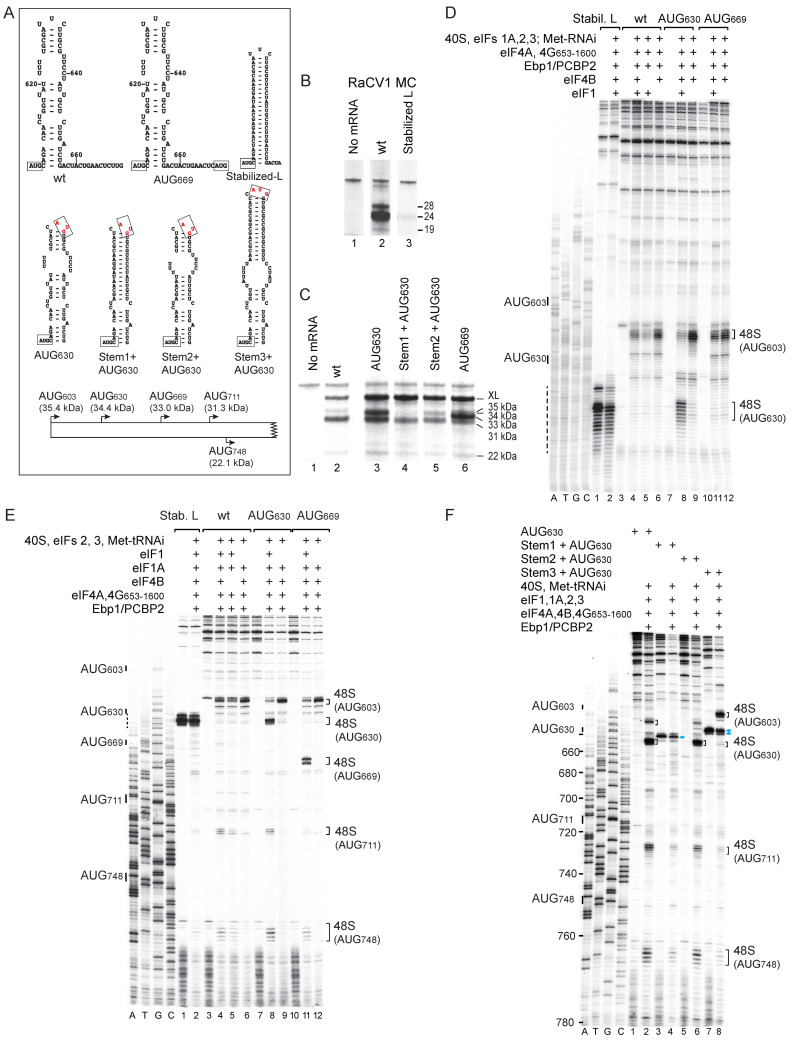
Influence of AUG codons and structural elements inserted downstream of the Yn-Xm-AUG motif on RaCV1 IRES-mediated initiation of translation. (**A**) Upper and middle panels: sequence and structure of IRES domain L and variants thereof, showing base-pairing and the positions of inserted codons AUG_630_ and AUG_699_. Lower panel: schematic representation of the coding potential of RaCV1 mRNAs, showing initiation codons present in different mutants and the molecular weights of corresponding translation products. (**B**,**C**) In vitro translation of (**B**) monocistronic and (**C**) dicistronic mRNAs containing the indicated wild-type and mutant IRESs. Panels are annotated to show translation products resolved by SDS-PAGE. (**D**–**F**) Toeprint analysis of 48S complexes formed on (**D**,**E**) wild-type and (**D**–**F**) mutant IRES-containing mRNAs by incubation with 40S subunits, Met-tRNA_i_^Met^, eIFs, Ebp1 and PCBP2 as indicated. Panels are annotated to show the positions of potential initiation codons on the left and of toeprints corresponding to 48S complexes on the right, and (**D**,**E**) with dashed lines to indicate RT stops induced on mRNA in the absence of factors by the stabilization of domain L. A dideoxynucleotide sequence generated with the same primer was run in parallel on each gel (lanes A, T, G, C) to show the corresponding wild-type sequence. Separation of lanes in panels (**B**,**C**) by white lines indicates that they were juxtaposed from the same gel.

The RaCV1 IRES has properties that are distinct from those of the GTCV IRES, and we therefore selected it for a series of complementary experiments. Domain L of the RaCV1 IRES is located immediately downstream of the Yn-Xm-AUG motif rather than between domain J and this motif (as in GTCV) and unlike GTCV, the RaCV1 genome contains an overlapping ORF (ORF1*) within ORF1. The translation of ORF1 might start at UUUAUG_603_C, which is part of the Yn- Xm-AUG motif, or downstream at UCAAUG_711_G. In the wild-type genome and in the monocistronic mRNAs used here, the translation of ORF1* could start at AGGAUG_748_U, but dicistronic mRNAs containing the RaCV1 5′UTR and proximal coding sequence linked to a reporter ORF contained an adventitious C_729_G substitution that introduced an upstream AUG codon (AGCAUG_7__29_A), and initiation at this codon would yield an N-terminally extended 22.1 kDa form of ORF1* (Figure 4A). The translation of monocistronic mRNA in RRL yielded a weak 28 kDa product, a prominent 24 kDa product and a weak 19 kDa product, predicted to initiate at AUG_603_, AUG_711_ and AUG_748_, respectively (Figure 5B, lane 2). In vitro translation of dicistronic mRNAs in RRL yielded a prominent 42 kDa product that corresponded to the XL product of the upstream ORF, weak 35 and 22 kDa translation products and a prominent 31 kDa product (Figure 4B, lane 2). The last three polypeptides correspond to the predicted products of translation of RaCV1 ORF1 and ORF1*. The RaCV1 5′UTR therefore functions as an IRES.

Substitutions that improved the context of AUG_603_ from UUUAUGC to ACCAUGG in dicistronic mRNAs significantly increased the prominence of the 35 kDa translation product while reducing that of the 31 and 23 kDa products (Figure 4B, lane 3). The substitution of AUG_603_ abolished translation of the 35 kDa product, whereas the elimination of AUG_711_ abolished translation of the 31 kDa polypeptide and strongly enhanced translation of the 23 kDa ORF1* product that resulted from initiation at AUG_729_ (Figure 4B, lanes 4, 5). In in vitro reconstitution experiments, 48S complexes assembled on monocistronic RaCV1 mRNA at AUG_603_, AUG_711_ and AUG_748_ (Figure 4D, lanes 1–5), which is consistent with the assignment of these triplets as initiation codons. Changes in the prominence of the translation products resulting from the improvement of AUG_603_ context, insertion of AUG_729_ or elimination of AUG_603_ or AUG_711_ are consistent with an initiation mechanism involving scanning after ribosomal attachment at or upstream of AUG_603_.

### 3.5. The Mechanism of Initiation on the RaCV1 IRES

To characterize the mechanism of initiation on the RaCV1 IRES in detail, the process was reconstituted in vitro from individual purified components of the translation apparatus. The activity of the RaCV1 IRES in mediating initiation in RRL (Figure 4B) indicates that the canonical eIFs and the ITAFs present in RRL are sufficient to support initiation on the IRES.

Initiation on different picornavirus type 2 IRESs is enhanced by Ebp1 and/or the pyrimidine tract-binding protein PTB [32,33,34,35,36,59]. We tested the influence of these and other ITAFs individually and in combination on initiation on the RaCV1 IRES. DHX29, which is required for initiation on type 5 IRESs (e.g., [28,29]), had no effect, and nPTB inhibited initiation complex formation, whereas initiation at AUG_603_, AUG_711_ and AUG_748_ was enhanced by Ebp1, by PCBP2 (Figure 4C, lanes 2–5) and synergistically by Ebp1 and PCBP2 (Figure 4D, lanes 2–5). Incubation of the IRES with unmodified wt Ebp1 and with a panel of Fe(II)BABE-modified wt and mutant forms of Ebp1 yielded a toeprint at nt 360 (Figure 4F), indicating that Ebp1 bound specifically and independently to subdomain Id. 48S complex formation at AUG_603_ and AUG_711_ of ORF1 and at AUG_748_ of ORF1* was dependent on the presence of Met-tRNA^Met^_i_ (e.g., Figure 4E, lane 5). AUG_603_ has poor nucleotide context (UUUAUGC), and its improvement (to ACCAUGG) strongly enhanced initiation at this codon (Figure 4E, lanes 1, 4). The translational competence of the in vitro assembled 48S complexes was assayed using RaCV1 IRES-linked mRNA with substitutions that placed UAA termination codons 9nt downstream of AUG_603_ and AUG_711_. The addition of 60S subunits, eIF5, eIF5B, eEF1H, eEF2 and total aminoacylated tRNAs led to the formation of elongation-competent 80S ribosomes that stalled on the UAA_612_ and UAA_720_ stop codons, forming pre-termination complexes (Figure 4G). AUG_748_ was not followed by a nearby in-frame stop codon, so that although 48S complexes that had assembled on this codon were able to undergo subunit joining and elongation in these conditions, as evident by the disappearance of corresponding stops downstream of AUG_748_ (Figure 4G, lane 3), these elongation complexes did not form pre-TCs that were detectable by toeprinting.

The assembly of 48S complexes at AUG_603_ was impaired by the omission of eIF1A, whereas the omission of eIF1 with or without eIF1A enhanced their formation at this codon (Figure 4H). The formation of 48S complexes on AUG_711_ and particularly at AUG_748_ was strongly reduced by the omission of either or both factors (Figure 4I). These data implicate eIF1A in promoting the recruitment of 43S complexes to the IRES and eIF1 in their movement to initiation codons downstream of the attachment site, by promotion of their adoption of a scanning-competent conformation and by discrimination against initiation at AUG_603_ because of its unfavorable nucleotide context [53,63,83].

The presence of domain L between AUG_603_ and the downstream AUG_711_ and AUG_748_ raises the question of whether 43S complexes unwind this domain completely to scan downstream or if they can also bypass this domain and be loaded downstream of it. To investigate this, AUG codons with the context AUCAUGU were inserted at the apex of domain L (AUG_630_) or downstream of it (AUG_669_) (Figure 5A). Both codons were recognized and used efficiently in RRL (Figure 5C, lanes 3, 6) and in in vitro reconstituted reactions (Figure 5D, lane 8; Figure 5E, lanes 8, 11). Notably, initiation at these and downstream codons (AUG_711_, AUG_748_) was strongly dependent on eIF1, and its omission led to the accumulation of 48S complexes at AUG_603_ (Figure 5D, lanes 8, 9, 11, 12; Figure 5E, lanes 8, 9, 11, 12).

The stabilization of domain L (ΔG = −24.4 kcal/mol) (Figure 5A) almost completely abrogated IRES-mediated translation in RRL (Figure 5B, lane 3) and initiation in in vitro reconstituted reactions (Figure 5D, lanes 1, 2). Note that in these experiments, the stabilized domain L arrested reverse transcriptase, yielding stops at the base of this domain (Figure 5D, lanes 1, 2; Figure 5E, lanes 1, 2). The loss of function could be due to a failure of 48S complexes to bind to the IRES or to an inability to unwind the hairpin and scan downstream. An AUG codon placed at the apex of a stabilized domain L (“Stem1 + AUG_630_”) (Figure 5A) was not utilized in RRL or in in vitro reconstituted reactions (Figure 5C, lanes 2–4; Figure 5F, lanes 1–4), but a low level of initiation occurred at AUG_711_, suggesting that a small proportion of ribosomes bypasses domain L. The interruption of base-pairing in the helix in [Stem2 + AUG_630_] mRNA enabled initiation to occur at the AUG_630_-equivalent codon (Figure 5C, lanes 3, 5; Figure 5F, lanes 1, 2, 5, 6), but its utilization was almost completely eliminated if it was sequestered in a stable hairpin placed on top of a stem with alternating bulges and helical elements ([Stem3 + AUG_630_]) (Figure 5A). Initiation on AUG_603_ in this mRNA was markedly higher than on [Stem2 + AUG_630_] mRNA containing AUG_630_ without the stable apical hairpin (Figure 5F, lanes 8), indicating that 43S complexes can bind to the Yn-Xm-AUG motif when the proximal stem of domain L contains discontinuities, but subsequent scanning is blocked by the stable apical hairpin. It is well established that initiation by scanning ribosomal complexes on weak initiation codons is enhanced by stable structures optimally placed ~14 nucleotides downstream [84].

### 3.6. Conserved Structures and Interactions in RaCV1 IRES Function

**Figure 6:**
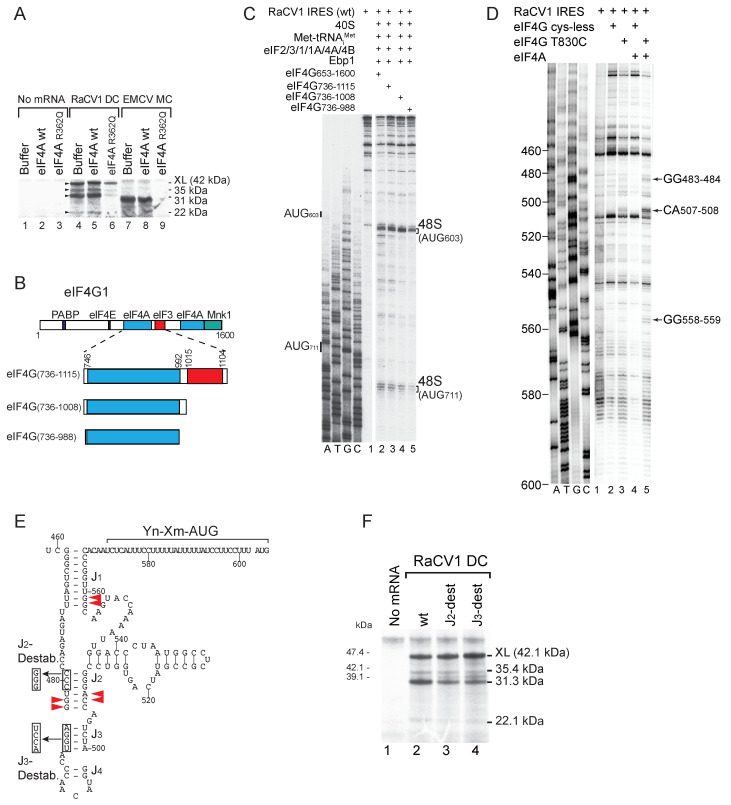
Requirement for subunits of eIF4F for initiation on the RaCV1 IRES. (**A**) Influence of recombinant eIF4A (wt) and eIF4A^R362Q^ on translation in RRL initiated on RaCV1 (lanes 4–6) and EMCV IRESs (lanes 7–9), (**B**) Schematic representation of full-length and truncated fragments of eIF4G1, showing factor binding sites. (**C**) Toeprinting analysis of 48S complex formation on RaCV1 MC mRNA in the in vitro reconstituted system in the presence of 40S subunits, Met-tRNA_i_^Met^, Ebp1, eIFs 1, 1A, 2, 3, 4A, 4B, and eIF4G_736–1599_, eIF4G_736–1115_, eIF4G_736–1108_ or eIF4G_736–988_, as indicated. Toeprints from 48S complexes assembled on AUG_603_ and AUG_711_ codons are indicated on the right. Separation of lanes by a white line indicates that they were juxtaposed from the same gel. (**D**) Primer extension analysis of directed hydroxyl radical cleavage of the wt RaCV1 IRES from Fe(II)-tethered eIF4G_736–1115_-[T830C], in the presence/absence of eIF4A, as indicated. A dideoxynucleotide sequence generated with the same primer was run in parallel on gels shown in panels (**C**,**D**) (lanes A, T, G, C) to show the corresponding wild-type sequence. The white lines in panel (**D**) indicate that the exposure of DHRC lanes 1–5 differed from that of the sequencing lanes A, T, G, C, but that sets of lanes were juxtaposed from the same gel. (**E**) Model of the J-K domain and adjacent Yn-Xm-AUG motif of the RaCV1 IRES, annotated to show structural elements (as in Figure 1B), sites of hydroxyl radical cleavage from Cys_830_ on eIF4G_736–1115_ (indicated by blue arrowheads) and destabilizing substitutions in mutant J_2_-dest and J_3_-dest RaCV1 IRESs. (**F**) In vitro translation of dicistronic mRNA containing the wt RaCV1 IRES (lane 2), J_2_-dest and J_3_-dest mutant IRESs (lanes 3, 4). The panel is annotated to show translation products resolved by SDS-PAGE.

The sensitivity of RaCV1 IRES-mediated translation to inhibition by dominant-negative eIF4A^R362Q^ (Figure 6A) implicates the eIF4G/eIF4A complex in this process as for initiation on GTCV and other type 2 IRESs (Figure 3B). The 48S complexes formed on the RaCV1 IRES in in vitro reconstituted reactions that included eIF4G_653–1600_ and eIF4A in place of eIF4F (Figure 5E), indicating that initiation is independent of eIF4E and of the N-terminal region of eIF4G that includes the eIF4E binding site. eIF4G also contains an eIF3-binding domain and two eIF4A-binding domains (Figure 6B).

To establish whether they are required to support initiation on the IRES, eIF4G_653–1600_ was substituted by recombinant fragments of eIF4G. The 48S complex formation at AUG_603_ was unaffected by deletion of the C-terminal eIF4A-binding domain and of the eIF3-binding site (a.a. 1015–1104) but was reduced by further C-terminal truncation to a.a. 989, which was likely due to the weakened binding of eIF4G to eIF4A (Figure 6C, lanes 2–5). Initiation at AUG_711_ was more strongly affected than at AUG_603_ by these deletions. These elements of eIF4G therefore contribute to the scanning competence of 43S complexes following their recruitment to this IRES. The omission of eIF4B impaired initiation, particularly at AUG_711_ and AUG_748_ (Figure 5E, lanes 4, 5). eIF4B therefore also contributes to the scanning competence of 43S complexes.

Initiation on type 2 IRESs relies on the specific binding of eIF4G’s central domain to the J-K domain, which is enhanced by eIF4A [30,55]. We used directed hydroxyl radical cleavage (DHRC) to determine whether eIF4G binds to the RaCV1 IRES in the same manner. In this approach, Fe(II) is site-specifically tethered by the linker 1-(*p*-bromoacetamidobenzyl)-EDTA (BABE) to a cysteine residue on the surface of an RNA-binding protein. Hydroxyl radicals generated by ascorbic acid/H_2_O_2_ treatment cleave mRNA with an intensity that is distance dependent. Cleavage is detected by primer extension inhibition and is strong within 22 Å, has medium intensity between 12 and 36 Å and is weak within 20–44 Å of the tethering position [85]. We used a BABE-linked variant of eIF4G_736–11116_ containing a single cysteine residue (in place of Thr-830) that binds type 2 IRESs in a known orientation [21,23]. Primer extension revealed cleavage at GG_483–4_, CA_507–8_ and GG_558–9_ that was strongly enhanced by eIF4A (Figure 6D) and that mapped to positions in the J-K domain that coincide directly with the strongest cleavages induced by this BABE-modified eIF4G variant in the EMCV IRES [21]. They overlap a bipartite motif near the apex of domain J that constitutes part of the eIF4G binding site in type 2 IRESs and is important for their function [21,22,23]. Consistently, destabilizing substitutions in the apical helices of domain J (Figure 6E) moderately impaired RaCV1 IRES-mediated translation (Figure 6F).

**Figure 7:**
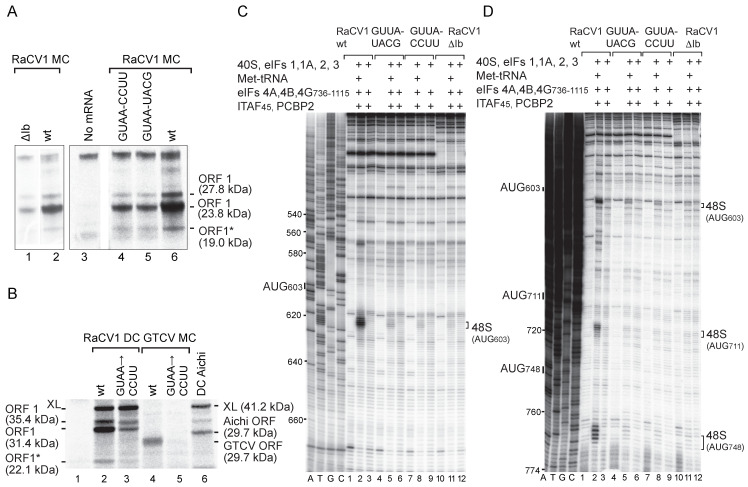
Influence of the GNRA tetraloop at the apex of subdomain Ib on RaCV1 IRES function. (**A**,**B**) In vitro translation of monocistronic and dicistronic mRNAs containing the indicated wild-type and mutant GTCV and RaCV1 IRESs. Panels are annotated to show translation products resolved by SDS-PAGE. Separation of lanes in panel (**A**) by a white line indicates that they were juxtaposed from the same gel. (**C**,**D**) Toeprint analysis of 48S complexes formed on wild-type and mutant IRES-containing mRNAs by incubation with 40S subunits, Met-tRNA_i_^Met^, initiation factors, Ebp1 and PCBP2 as indicated. Panels are annotated to show the positions of initiation codons on the left of toeprints corresponding to 48S complexes on the right. A dideoxynucleotide sequence generated with the same primer was run in parallel on each gel (lanes A, T, G, C) to show the corresponding wild-type sequence.

Analysis of another conserved element in the IRES showed that substitutions in the apical GNRA tetraloop of subdomain Ib in monocistronic and dicistronic mRNAs and deletion of Ib in monocistronic mRNA strongly impaired initiation at AUG_603_, AUG_711_ and AUG_748_ in RRL (Figure 7A, lanes 1, 2, 4, 5; Figure 7B, lane 3). Consistently, these mutations strongly impaired 48S complex formation at these codons in in vitro reconstituted initiation reactions (Figure 7C,D).

### 3.7. Specific Binding of Ebp1 to Calicivirus Type 2 IRESs

**Figure 8:**
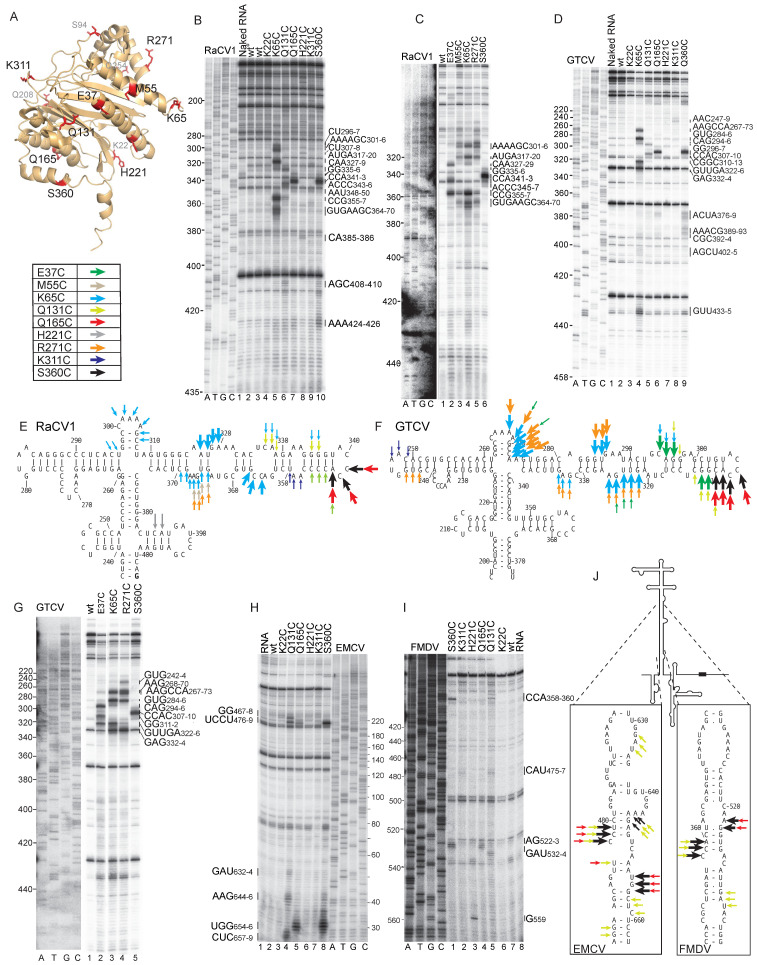
Interaction of Ebp1 with type 2 IRESs. (**A**) Upper panel: schematic representation of Ebp1 showing the positions of residues substituted by cysteine residues in individual mutants. Residues labeled in large black font induced cleavage of the bound IRES; residues labeled in smaller gray font did not. Lower panel: the color scheme used to indicate residues that induced cleavage on models of (**E**) the RaCV1 IRES, (**F**) the GTCV IRES and (**J**) EMCV and FMDV IRESs. (**B**–**D**,**G**–**I**) primer extension analysis of DHRC of (**B**,**C**) the RaCV1 IRES, (**D**,**G**) the GTCV IRES, (**H**) the EMCV IRES and (**I**) the FMDV IRES from Fe(II)BABE-linked Ebp1 mutants. Sites of cleavage are indicated (**B**–**D**,**G**,**I**) on the right, (**H**) on the left and (**E**,**F**,**J**) mapped onto IRES models. Reference lanes A, T, G, C on each panel show the appropriate viral sequence generated with the same primer, except in (**H**), which shows the FMDV sequence and is numbered to indicate the number of residues from the 3′-end of the primer. Separation of lanes by white lines in panels (**C**,**G**) indicates that they were juxtaposed from the same gels; the intensity of sequence lanes in (**C**) was increased.

Initiation on GTCV and RaCV1 IRESs uses overlapping sets of ITAFs: PTB enhances GTCV IRES function but is not required by the RaCV1 IRES, whereas initiation on the RaCV1 IRES is enhanced by PCBP2 (Figure 3G and Figure 4C,D). Initiation on both IRESs requires Ebp1, which was first identified as an ITAF that enhances the activity of the type 2 FMDV IRES [32]. An important step in elucidating how Ebp1 promotes initiation on GTCV and RaCV1 IRESs is to determine how it interacts with them. We employed directed hydroxyl radical cleavage to determine the binding site and the orientation of bound Ebp1 on this and other IRESs. If the structure of a Fe(II)BABE-modified protein is known, then cleavage at multiple sites enables the position and orientation of the protein relative to the RNA ligand to be established. Ebp1 consists of two anti-parallel β-sheets, each flanked by a pair of α-helices (Figure 8A) [35], and it adopts different conformations on the ribosome (e.g., [86,87,88]). It binds to the tunnel exit on the 60S subunit, and in one of the several reported structures [86], it is held in place by interaction with the ribosomal RNA expansion segment ES27L. However, it is important to note that Ebp1’s role in promoting 48S complex formation does not involve the 60S subunit.

The native cysteine residues in Ebp1 could not be substituted to yield a cysteine-less variant without loss of activity, but the incubation of wt Ebp1 with Fe(II)BABE) did not induce the cleavage of type 2 IRESs (Figure 8B, lane 3), suggesting that native cysteine residues in it are not surface-exposed and thus that cysteines could be placed at surface-exposed locations for DHRC experiments. We generated a panel of Ebp1 moieties with additional cysteine residues at positions 22, 37, 55, 65, 94, 131, 165, 208, 22, 254, 271, 311 or 360 (Figure 8A). Fe(II)BABE-linked cysteine residues at positions 37, 55, 65, 131, 165, 271 and 360 induced strong cleavage in the central and apical elements of subdomain Id of the RaCV1 IRES (Figure 8B,C; summarized in Figure 8E). Weaker, more dispersed cleavages mapped to the central stem of domain I.

The principal Ebp1 binding site on the RaCV1 IRES corresponds to an element that occurs in all avian calicivirus type 2 IRESs. The patterns of cleavage of GTCV and RaCV1 IRESs by Fe(II)-BABE-derivatized Ebp1 were very similar (compare Figure 8B–D,G), indicating that it binds to them at equivalent locations and with an identical orientation (Figure 8E,F). However, this binding site is not present in EMCV or FMDV IRESs even though they both bind to Ebp1 [35]. DHRC showed that Fe(II)-BABE-derivatized Ebp1 induced cleavage in the irregular stem of domain I of EMCV and FMDV IRESs with some weaker cleavages mapping to the junction of I, J-K and L domains (Figure 8H,I; summarized in Figure 8J). Notably, the cleavage of EMCV and FMDV IRESs was induced most effectively by residues that induced the strong cleavage of RaCV1 and GTCV IRESs, indicating that the same surface of Ebp1 binds to these IRESs.

**Figure 9:**
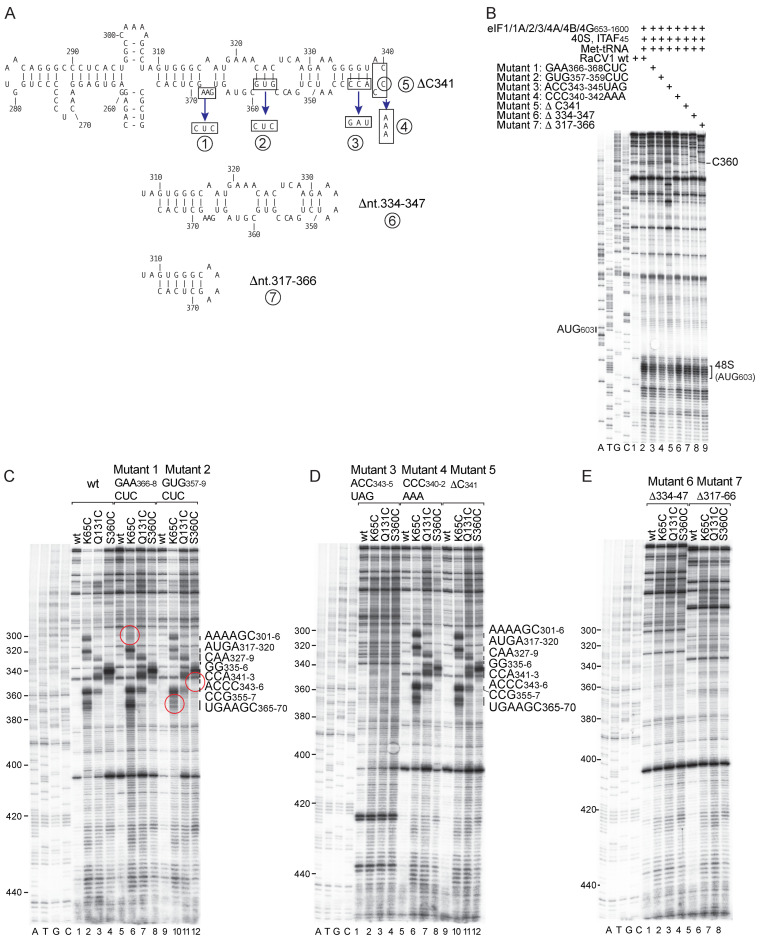
Influence of mutations in RaCV1 IRES subdomain Id on binding of Ebp1 assessed by directed hydroxyl radical probing and IRES activity. (**A**) Schematic model of RaCV1 IRES subdomain Id showing the substitutions/deletions in mutants 1–5 (upper panel) and residual predicted structure in deletion mutants 6 (middle panel) and 7 (lower panel). (**B**) Toeprint analysis of 48S complexes formed on wild-type and mutant IRES-containing mRNAs on incubation with 40S subunits, Met-tRNA_i_^Met^, initiation factors, Ebp1 and PCBP2 as indicated. Panels are annotated to show the positions of initiation codons on the left and of toeprints corresponding to 48S complexes on the right. (**C**–**E**) Primer extension analysis of directed hydroxyl radical cleavage of ((**C**) lanes 1–4) wt and (**C**–**E**) mutant RaCV1 IRESs from the indicated Fe(II)-tethered Ebp1 variants. Sites of cleavage are indicated on the right. (**C**) Circles show sites of defective cleavage associated with specific IRES mutations. A dideoxynucleotide sequence generated with the same primer and (**B**,**D**,**E**) wild-type and (**C**) [ACC_343–5_UAG] mutant IRESs was run in parallel on each gel.

The similarity in patterns of Ebp1-directed hydroxyl radical cleavage of the RaCV1 and GTCV IRESs is evidence for the specificity of binding. To characterize the functional importance of binding, we compared DHRC by Ebp1 and 48S complex formation on a panel of mutant RaCV1 IRESs with substitutions or deletions in subdomain Id (Figure 9A).

Levels of DHRC and initiation activity on these mutants correlated well: mutations 4 and 5 had little or no effect on cleavage (Figure 9D, lanes 5–12) or on the level of 48S complex formation (Figure 9B, lanes 6, 7), mutations 3, 6 and 7 abrogated cleavage (and therefore, binding) (Figure 9D, lanes 1–4; Figure 9E, lanes 1–8), and they supported 48S complex formation at up to only 50% of the wild-type level (Figure 9B, lanes 5, 8, 9), whereas mutations 1 and 2 exhibited site-specific defects in cleavage (Figure 9C, lanes 5–12) and mild reductions in the level of 48S complex formation (Figure 9B, lanes 3, 4).

## 4. Discussion

Caliciviruses use various mechanisms to maximize the utilization of their coding capacity, including (i) synthesis of a polyprotein that is cleaved to yield individual functional proteins, (ii) synthesis of subgenomic mRNA to allow the independent expression of structural proteins, and (iii) substoichiometric translation of a downstream ORF by a termination–reinitiation mechanism promoted by elements of the viral mRNA. We have now identified examples of another mechanism: translation of overlapping ORFs. This mechanism had previously been reported for an alternative coding region within ORF2 in the subgenomic mRNA of noroviruses [8,9]. The examples identified here are exemplified by translation of the ORF1 and ORF1* moieties of RaCV1 and *Caliciviridae* sp. Isolate CAL/PB27-SI31/Switzerland/2019.

The translation of ORF1* in the first group initiates downstream of the ORF1 initiation codon and yields a 239–317 a.a.-long polypeptide, whereas ORF1* in the second group initiates upstream of the ORF1 initiation codon and encodes a ~200 a.a.-long polypeptide. There are several precedents for IRES-mediated initiation at multiple initiation codons, either in-frame (as in FMDV), leading to the synthesis of an N-terminally extended polyprotein [25,79], or in overlapping reading frames, e.g., mediated by the type 1 poliovirus IRES [89,90], by the type 2 IRES of Theiler’s murine encephalomyelitis virus (leading to synthesis of the L* protein) [26] and likely by Cosavirus, Hunnivirus, Rosavirus and Sicinivirus type 2 IRESs [90]. Initiation at AUG codons downstream of the RaCV1 IRES involves scanning after IRES-mediated recruitment of the 43S complex and is dependent on eIF1 and eIF1A (Figure 4H). The nucleotide context of the ORF1 initiation codon in the RaCV1 group is in many instances suboptimal, so that it would be bypassed by an appreciable proportion of scanning ribosomes, ensuring the translation of ORF1*. The function of ORF1* is unknown, and it does not have significant sequence or structural homology with proteins of known function. Consistent with other polypeptides encoded by alternative reading frames [91], ORF1* moieties are predicted to be largely unstructured and might therefore function by binding to viral or cellular proteins. For example, the TMEV L* protein binds to RNAse L, an effector in the interferon antiviral response, and prevents its activation [92,93].

The exploitation of overlapping ORFs described above contributes to maximizing utilization of the coding capacity of calicivirus genomes. The presence of an IRES to promote translation of the overlapping ORFs potentially enables the virus to sustain the translation of both ORFs in circumstances that would impair cap-dependent translation. Calicivirus infections lead to the shut-off of cellular translation that is associated with changes in the translation apparatus, including cleavage of the poly(A) binding protein PABP, dissociation of eIF3 from eIF4G, and phosphorylation of eIF4E [14,94,95]. Notably, the EMCV IRES is less dependent on PABP for translation than cellular mRNAs [96], and its function is enhanced by dephosphorylation/activation of the regulatory eIF4E-binding protein (4E-BP) and by its overexpression [97], whereas the translation of cellular mRNAs and several caliciviral mRNAs is inhibited by 4E-BP [98]. Interestingly, the translation of calicivirus mRNAs that depend on the VPg-eIF4E interaction is also inhibited by 4E-BP [11,99], suggesting that the acquisition of a type 2 IRES by a calicivirus genome may alter its ability to sustain translation in specific stress conditions, resulting in a gain of function. As noted here for the RaCV1 IRES and elsewhere for the EMCV IRES [55], initiation on type 2 IRESs is not dependent on the eIF4G–eIF3 interaction. Taken together, these observations suggest that the presence of IRESs in calicivirus genomes, likely the result of horizontal gene transfer, may enhance their ability to usurp the cellular translation apparatus and to target cellular translation in a manner that does not impair viral translation.

Biochemical analysis revealed similarities and differences between the mechanism of initiation on calicivirus type 2 IRESs and on the canonical EMCV, FMDV and TMEV type 2 IRESs. The calicivirus IRESs require the same set of canonical initiation factors for 48S complex formation as these picornavirus IRESs [27,30,32,33,53,59]. The process similarly involves the binding of eIF4G to the JK domain, and it also does not depend on the eIF4G–eIF3 interaction ([55]; Figure 6), suggesting that another interaction must be responsible for directing association of the IRES with the 43S complex. However, eIF4G’s eIF3-binding domain enhanced initiation at RaCV1 codons downstream of the Yn-Xm-AUG motif, possibly indicating that the eIF3-eIF4G interaction is important during subsequent scanning, for example to promote processivity. Scanning also required eIF1, as noted previously for scanning after the recruitment of 43S complexes to type 1 IRESs and the type 2 FMDV IRES [27,31]. Our analysis shows that as in picornavirus type 2 IRESs, the eIF4G-binding elements in domain J [21,22], the conserved apical region of domain I (including the GNRA tetraloop) [16,17,18,19] and the Yn-Xm-AUG motif are all important for the function of calicivirus type 2 IRESs. Notably, despite its conservation and importance, the function of the tetraloop remains unknown.

Differences in the ITAF requirements of different type 2 IRESs have been noted previously, exemplified by the Ebp1-dependence of the FMDV IRES but not of the EMCV and TMEV IRESs [32] and the conditional PTB-dependence of EMCV IRESs, the latter being determined either by mutations in the IRES or by its linkage to different heterologous reporters [100]. Ebp1 dependence is likely also conditional, because the affinity of its binding to EMCV and FMDV IRESs is comparable despite their different requirements for this factor [35]. ITAF requirements on calicivirus type 2 IRESs also varied: the activities of both GTCV and RaCV1 IRESs were enhanced by Ebp1, but the GTCV IRES was stimulated by PTB, whereas initiation on the RaCV1 IRES was enhanced by PCBP2. A requirement for PCBP2 by type 2 IRESs has not previously been reported, although it binds specifically to the EMCV and FMDV IRESs without contributing to their function [101]. It may be that its function is also conditional, compensating for an activity or structural characteristic that is suboptimal in calicivirus type 2 IRESs but not in EMCV or FMDV IRESs. Whether PCBP2 binds to the apex of domain I in an analogous manner to its interaction with the apical tetraloop-containing region of domain IV in type 1 IRESs [31,100,102,103,104] remains to be determined. The function of Ebp1 in IRES-mediated initiation also remains obscure. The results of DHRC experiments showed that it binds directly to a conserved location on calicivirus type 2 IRESs that differs from the binding site on EMCV and FMDV IRESs. Mutations in the RaCV1 IRES that abrogated binding (assayed by DHRC) led to a partial loss of function. Interestingly, the silencing of Ebp1 expression in human, bovine, canine and porcine kidney cells resulted in a similar partial impairment of FMDV IRES activity [35,36]. Ebp1 is therefore important but not absolutely essential: in light of the differences in its binding site of calicivirus and picornaviruses IRESs, its function may, like other ITAFs, be to promote adoption by IRESs of an active conformation [105].

## Figures and Tables

**Table 1:** Classes of viral IRES.

IRES Class	Representative Member	Virus Family
1	Poliovirus	*Picornaviridae*
2	Encephalomyocarditis virus	*Picornaviridae*
	Foot-and-mouth disease virus	*Picornaviridae*
	*Caliciviridae* sp. isolate yc-13 “Red-crowned crane calicivirus”	*Caliciviridae*
3	Hepatitis A virus	*Picornaviridae*
4	Hepatitis C virus	*Flaviviridae*
	Ruddy turnstone calicivirus A	*Caliciviridae*
5	Aichivirus	*Picornaviridae*
	*Caliciviridae* sp. isolate hwf182cal1	*Caliciviridae*
6	Cricket paralysis virus	*Dicistroviridae*

## Data Availability

The original data presented in the study are openly available in Mendeley Data at https://data.mendeley.com/drafts/9mxx48n3sc.1 from 1 September 2024.

## Supplementary Materials

The following supporting information can be downloaded at https://www.mdpi.com/article/10.3390/v16091413/s1. Supplementary Figure S1. Sequence alignment of the conserved core of calicivirus type 2 IRESs. Supplementary Figure S2. Aligned ORF1* sequences from *Caliciviridae* sp. isolate yc-13 and related caliciviruses. Supplementary Figure S3. Aligned ORF1* sequences from *Caliciviridae* sp. isolate CAL/PB27-SI31/Switzerland/2019 and related caliciviruses. Supplementary Figure S4. (A) Aligned ORF1* sequences from *Caliciviridae* sp. isolate cftwhg06cal1, Goose calicivirus isolate MA/CHN/2018 and Goose calicivirus isolate H146, (B) Aligned ORF1* sequences from Duck calicivirus isolates MW20 and NC-19BV-45-k77-747816, (C) ORF1* sequence from *Caliciviridae* sp. isolate ybb044cal01nc. Supplementary Figure S5. Open reading frames (ORFs) upstream of or overlapping ORF1 in avian calicivirus genomes.
