# Supplementary material for: Translation of Overlapping Open Reading Frames Promoted by Type 2 IRESs in Avian Calicivirus Genomes
Source: Viruses. 2024 Sep 4;16(9):1413. doi: 10.3390/v16091413 (PMC11436067; doi:10.3390/v16091413)
Supplement: Supplementary file 1 [file viruses-16-01413-s001.zip › viruses-3155249-supplementary.pdf]

Supplementary Figure S1. Sequence alignment of the conserved core of calicivirus type 2 IRESs

Yn Yn

Domain H

```
Duck                  171  CCUAUACACCUGGCA-----CCACUUUU----GUGGUCUCGCUG--GGUGAAGGCCCC  217
Duck_NC-19BV          174  GUUAUACACCCGACA-----CCAUUUUU----GUGGUCUCGUUG--GGUGAAGGCCCC  220
Grey Teal             101  GCUAUACACAUCCCCUG----CACUCUU-------UGCUCUGGG-AUGUGAAGGCCCC  146
Trumpeter              81  GCUAAACACAGCCCAAG----CGUGCUU---UGCUUCUCGGGC--UGUGAAGACCCC  128
Caliciviridae361bR    192  CAUUGACAUAUGGCCC-----CACGCUU---GUGGAUCUUCUGCCAUAUGAUGGACCC  241
Chicken_NLD_036       214  CAUUGACACAUGGCCC-----CACGCUU---GUGGAUCUCUAGCCAUGUGAUGGACCC  263
CAL/PB30-SII34        121   AUUGACACAUGGCCC-----CACGCUU---GUGGAUCUCUAGCCAUGUGAUGGACCC  170
RaCV1                 128   CUAUAAUGAGCAACCGAACCCCGUCUU-GGGGUGUACUCUCGGUACUCAAUGGACCC  183
Avocet                167  UCUAACGUAUCUCCCUCCCCACACUCUUUGUGGGAUGCUCUGGG-AGACGAUGGACCC  223
Wilkes                173  UUUAAUGAUGUUG--GCACCGCAACCUU----GCGGCCUUGCUGGUAUCAAUGGACCC  224
Pink-eared duck       188   CUAAGG-UGGCU--GGAACAGCAACUU----GCUGUCUCUCUGGCUGCCAUGGAUCC  237
xfoti                  11   UUAAGGCUGCUGG--CACCGCCGUCUU---GGCGGUCUCGCUGGCAUCCAUGGACCC  62
Duck PI202116          11   UUAAGGCUCCUGG--CACCUCCGACUU---GGAGGUCUCGCUGGGAUCCAUGGACCC  62
```

Domain I

```
Duck                  218  -AUUGGCCGUCUGG-UCACAGCACGGCCCAACCUAGUA-CCCACGAAC-GCACCCAACUGA  274
Duck_NC-19BV          221  -AUUAGCCGAGCUUCCACAAGCACGGCCCUAGUUAGUA-CCUGCGAAA-GCUACUGCGUGA  278
Grey Teal             147  -GUUGCUCUUGCAACGGCAAGUAUGGCGUAGCCUAGUA-CUGGCGAACGUCACUCAUGCGU  205
Trumpeter             129  GUCUCCCAGCAGUUUUUGCUAUAUGGCAUAAGCGCUGG--GGUCGAACGUUGGCAC-GCGU  186
Caliciviridae361bR    242  CCAACCACUUACUAGCUUAUGUACCCUAGUGAUCAGUACCUGCCGAAACU-GACACCGUGU  301
Chicken_NLD_036       264  CCACUCACUUGUUCGUUUAUGUACCAUUGUGAUCAGUACCUGCCGAAACU-GACAUCGUGU  323
CAL/PB30-SII34        171  CCACUCACUUGUUUGUUUAUGUACCAUUGUGAUCAGUACCUACCGAAACU-GACACCGUGU  230
RaCV1                 184  CAUUUAAGUAACCC--UCGCUGUUGAC-ACAGCGGCGGGUUUCCGAACCC-UUCUCCUGAU  240
Avocet                224  UAACUGACUAACCUU-GGUACGACGACACUGUCGUCGC-AUCACGAACACACUUCUUGUGU  282
Wilkes                225  CUACCACCUAACCC--ACUCACUUGACACAUGUGGUUCGCCCACGAAACCCCC-UUUGUGU  282
Pink-eared duck       238  AUAUUCUGUAACCC--UGUGCGUUGACGCGCAUGGAGUUACUCCGAACCCCC-UUUGCGU  295
xfoti                  63  UAACACUGUCAUCC--UGCCUGUUGACACAGGUAGGGCUAUGACGAACACCC-UUUUGUGU  120
Duck PI202116          63  UAACACUGUCACCC--UGCCUGUUGACACAGGUUGGGCUAUGACGAACACCC-UUUUGUGU 120
```

C-rich GNRA

```
Duck                  275  GCGUCGCGGCGUC-AUGAGGCGGGUGGAGCACCCCCCUGGUAACAGGUCCUCCCGGGCC  333
Duck_NC-19BV          279  GCGUCGCGGCGUC-AUAGAGCGGGUGGAAUCCCCCCCUGGUGACAGGAGCCUCCCGGGCC  337
Grey Teal             206  GUCUCGCGACGCGUAGUGAGGGUGUGGAAACCCCACGUGGUAACACGUGCCACACUGGCC  265
Trumpeter             187  GCGUCGCGGCACCAAUGCUGGGUGUGGUUACCCCACAUGGUAACAUGUGCCACACUGGCC  246
Caliciviridae361bR    302  GGCUCGCGCCACCUGCAGUGGGAGUGGAUCCCCCAUCUGGUAACAGGUGCCACUCUGCCC  361
Chicken_NLD_036       324  GGCUCGCGCCACCUGCAGUGGGAGUGGAACCCCCACUUGGUAACAAGUGCCACUCUGCCC  383
CAL/PB30-SII34        231  GGCUCGCGGCACCUGCAGUGGGAGUGGACUCCCCACUUGGUAACAAGUGCCACUCUGCCC  290
RaCV1                 241  GGCUCGCGGCAUCCCAGAGGAGUGAGGAACUCCCCCCUGGUAACAGGGCCCUCACUGGCC  300
Avocet                283  GGCUCGCGCCAUCCAAGAGGAGUUGGGAACUCCCCCCACGGUAACGUGGCCCAACUGGCC  342
Wilkes                283  GGCUAGCGCCAUCUGAGAGGAGUGCGGAACUCCCCCCACGGUAACGUGGCCCGCACUGGCC  342
Pink-eared duck       296  GGCUCGCGCCAUCCAAGAGGAGUGUGGAACCCCCCCACGGUAACGUGGCCCACACUGGCC  355
xfoti                 121  GGCUCGCGCCAUCCAAGAGGAGUAGGGAACUCCCCCCUGGUAACAGGGCCCCUACUGGCC  180
Duck PI202116         121  GGCUCGCGCCAUCCAAGAGGAGUGGGGAACUCCCCCCUGGUAACAGGGCCCCCACUGGCC  180
```

AAA

```
Duck                  334  ACAAGCCACGUGGGCAGGGCGAAGGUGAAGGAAGGGUCCCCCGACCCAACC-UCAA-GCCU  392
Duck_NC-19BV          338  AAAAGCCACGUGGGCAGGGCGAGAGCAAAGAAAGGGUCACCCGACCCAAUC-UCAA-ACUC  396
Grey Teal             266  AAAAGCCAAGUGGACAGGGUGAAUACUGCAGGGAGCUGUACCCACGGC-UCCUCUAAGUUG  325
Trumpeter             247  AAAAGCCAAGUGGGCAGGUUGGAAGCCUAAGGCAGCUGUACCCACGGC-UGCUCUAAGUGG  306
Caliciviridae361bR    362  AAAAGGCUAGUGUAUAGGACUGUCUACUGGUCG-GUUGUACCCACGGCCUGC-CUACUAGGC 421
Chicken_NLD_036       384  AAAAGGCUAGUGUAUAGGCUUGUCUACUGGUUG-GCUGUACCCACGGCCCGC-CUACUAGGC 350
CAL/PB30-SII34        291  AAAAGGCUAGUGUAUAGGUCUGUCUACUGGUUG-GCUGUACCCACGGCCCGC-CUACUAGGC 443
RaCV1                 301  AAAAGCCUAGUGGGCAAUGAAACACUCAAGAAA-GGGGUACCCACCCCAA--UCUGACCGUG 359
Avocet                434  UAAAGCCUAGUGUGCAAUCAACAUGCCUAGUCG-GCUGUACCCACGGCCUGG-CUUACGUAU 402
Wilkes                343  UAAAGCCUAGUGUGCAAGGACACAUGCUGGUCG-GUUGUACCCACGGCCUGC-CUACCAUG  401
Pink-eared duck       356  UAAAGCCUAGUGUGCAGGAUUGCUGCCUGGACG-GCUGUACCCACGGCCUGUCCUACGUAG  415
xfoti                 181  AAAAGCCUAGUGUGCAGGAAUUAAGUCUAGUCG-GCUGUACCCACGGCCUGG-CUUACGCUU 240
Duck PI202116         181  AAAAGCCUAGUGUGCAGGAAUUAAGUCUAGUCG-GCUGUACCCACGGCCUGG-CUUACGCUU 240
```

C-rich

```
Duck                 393  GAACCCG-CGUUCACCCUCAUGAUCAGGCAACCCAGCCUGGCGGUUGUUUGUUAGUAUUG 453
Duck_NC-19BV         397  GAACCCG-CGUUCACCUCUAUGAUCAGGCAACCCAGCCUGGCACUCUUAGUUAGUAACA 456
Grey Teal            326  AAGCCCG-AGUUCACCCACUACGUUGUGCUACCCCGCACAGCGCCUGUUUGACUAGUAUCC 385
Trumpeter            307  AAAACUG-CGUCCACCACAUUGGUUGUGUAACCCCAACACUGCGUGUGUCAGCUAGUACAC 366
Caliciviridae361bR 422  AUUCU-GCAGUACACCCUGCAGG-CGU-CAACCCCAGACAACACGUUGUUCAUUAGUACGU 479
Chicken_NLD_036      444  AUGCU-GCAGUACACCCUGCAGG-CGU-CAACCCCAGACAACACGUUGUUCAUUAGUACGU 501
CAL/PB30-SII34       351  AUGCU-GCAGUACACCCUGCAGG-CGU-CAACCCCAGACAACACGUUGCUCACUAGUACGU 408
RaCV1                360  CGUAGUGAAGCUCACCGC-UGGGACUCAUGAUCCC-GAUGAAUCAGGUUAGCUAGUACGA 418
Avocet               403  GUUGGUGAAGCUCACCGC-UUGGACUC-CGACCCCGGGAACACAAA--AGUGCUAGUACUG 459
Wilkes               402  UGUCCUGAAGCUCACCGC-UCAGACCU-CGAUCCCGGAGACACACU--GGGGCUAGUACUG 458
Pink-eared Duck      417  UUGUCCGAAGCUCACCGC-UUGGACUC-CGACCCCGGGAACGCAAC--UGGGCUAGUACGA 472
xfoti                241  AAGACCGAAGCUCACCGC-UUGGACUC-CGACCCCGGGAACACAAC--UGGGCUAGUAAUC 297
Duck PI202116        241  AAGACCGAAGCUCACCGC-UUGGACUC-CGACCCCGGGAACACAAC--UGGGCUAGUAAUC 297
```

Domain J- K

```
Duck                 453  GCGAAAAGCUGGCACCGACUGUUCCCAAGACCACCCAAUGGCAAGGGGGCUGGUGAGUAG 512
Duck_NC-19BV         457  GCGAAAGCAAGGCACCGACUUGCGUUAGUUCCACCUAAUGGCAAGGGGGUAGGUGAGUAG 516
Grey Teal            386  ACGAAACGCUACCCCCAGCUUGCCCGCGUGACUGACAACGGCACUGGGUUUGGCGAGUAG 445
Trumpeter            367  CCCAAACCUUUUCCCCAGUAGCAUCUUCUGCUUCUGGACGGCACUGGGUUUGGCGAGUAG 426
Caliciviridae361bR 480  GUGGG-AACGCUCUAGGAU-CAUCUGCUAAGUAUUUGUUUGGGUCUCCCCCGCUUAGUAG 537
Chicken_NLD_036      502  GUGGG-AACGCUCAAUGAU-CAUCUACUGAACAUUUGCGUGGGUCUCCCCCGCUCAGUAG 559
CAL/PB30-SII34       409  GUGGG-AACGCUCAAUGAU-CAUCUACUGAACAUUUGCGUGGGUCCCCCCCGCUCAGUAG 466
RaCV1                419  ACCCC-AAAGCUGGACCAC-CAACAGCCUGGGCC-UUAAAUGGUCGGGCUGAUUUAGUAG 475
Avocet               460  CUGUGGCUUCAGGACCACC-GUGUCCCAAGGCUGACAGUUAGCCCCCCCCGGCUUAGUAG 518
Wilkes               459  UGCGCGCCGCCUGGACCAC-CAAGUCCUUGGGCAGUGCGUAGGACUGCCAGCCUUAGUAG 517
Pink-eared duck      473  CUAGCUCUUUGUGAACCAC-CAACCCACAGGAAC-AGCAUAUGCUGUCCUGUUCUAGUAG 530
xfoti                298  CUAGCUCUCCCUGGACCAC-CAACUUGCAGGACC-AGCGUUAGCUGGUCUGUCUUAGUAG 355
Duck PI202116        298  CUAGCCUACCCUGGACCAC-CAACUUGCAGGACC-AGCGUUAGCUGGCCUGUCUUAGUAG 355
```

Bipartite motif

```
Duck                 513  AUGCCCUGCAGGUACCCUUU----CGGAUCUGAGCAGGGGCCCUUGACUAUACCGUUCCG 568
Duck_NC-19BV         517  AUGCCCAGGAGGUACCUCUAU--UGAGAUCUGACCUGGGGCCCUUGACUAUACCGUUCCG 574
Grey Teal            446  AUGCCCUGGAGGUACCCACU---CUGGAUCUGACCAGGGGCCCGUGACUAUACCGCUCCG 502
Trumpeter            427  AUGCCCUGGAGGUACCCUCU------GAUCUGACCAGGGGCCCGUGACUAUACCGUUCCG 480
Caliciviridae361bR 538  ACCCCCUGAAGGUACCCCUUAAAUGGGAUCUGAUCAGGGCCUGGUGACUAUACCGCUCCG 597
Chicken_NLD_036      560  ACCCCCUGGAGGUACCCCUUAACUGGGAUCUGACCAGGGCCUGGUGACUAUACCGCUCCG 619
CAL/PB30-SII34       467  ACCCCCUAGAGGUACCCCUUAAUUGGGAUCUGACUAGGGCCUGGUGACUAUACCGUUCCG 526
RaCV1                476  ACCCCCUGGAGGUACCCAACA---UGGAUCUGACCAGGGCCUGGUGACUAUGCCGCUCCG 532
Avocet               519  ACCCCCUGGAGGUACCCCUAA--CGGGAUCUGACCAGGGCCACUUGACUAUACCGAUCCG 576
Wilkes               518  ACCCCCAGCAGGUACCCCCUU----GGAUCUGAGCUGGGCCCUGUGACUAUACCGUUCCG 573
Pink-eared duck      531  ACCCCCUGGAGGUACCCUUCA--CAGGAUCUGACCAGGGCCUGGUGACUAUACCGCUCCG 588
xfoti                356  ACCCCCUGCAGGUACCCCGAA--AGGGAUCUGAGCAGGGCCUGGUGACUAUACCGCUCCG 413
Duck PI202116        356  ACCCCCUGCAGGUACCCCGAA--AGGGAUCUGAGCAGGGCCUGGUGACUAUACCGCUCCG 413
```

ASL

```
Duck                 569  GUAAUCAGGGGGUAAGAACCAUGAAAGCGCAGCCCCC      605
Duck_NC-19BV         575  GUAAUCAGGGGAUAAGAACCAUGAAAGCGCUACCCCC      611
Grey Teal            503  GUAAACCGGGUAUAAAAACCAUGAAAGUGCAAACCCA      539
Trumpeter            481  GUAAACCGGGUGUAAAAACCAUGAAAGUGCAAACCCA      517
Caliciviridae361bR 598  GUAAUCCCAGGUUAAAA-CCAUGAACGGCGGGGGGGG-     633
Chicken_NLD_036      620  GUAAUCCCAGGUUAAAA-CCAUGAACGCGGGGGG---      655
CAL/PB30-SII34       527  GUAAUCCCAGGUUAAAA-CCAUGAACGGCGGGGGGGG-     562
RaCV1                533  GUAAUCCCAGGUUAAAA-CCAUGAACGGUUGGCCC---     536
Avocet               577  GUAAUGAGUGGUUAAAA-UAAUGAACGGCUGGGGG---     610
Wilkes               574  GUAAUGCAGGGUUAGAA-AAAUGAACGGGUUGGCA---     607
Pink-eared duck      589  GUAAUCCCAGGUUAAAA-CCAUGAACGAAUAGGAC---     622
Goose_NC-19BV          1         CCAGGUUAAAA-CCAUGAACGAAUGGGCCCCA     31
xfoti                414  GUAAUCCCAGGUUAAAA-CCAUGAACGGAUAGGCC---     448
Duck PI202116        414  GUAAUCCCAGGUUAAAA-CCAUGAACGGAUGGGCC---     448
```

Supplementary Figure S1. Sequence alignment of the conserved core of calicivirus type 2 IRESs. Sequence alignment of elements corresponding to IRES domains H, I and J-K in the 5'UTRs of duck calicivirus MW20 ('Duck'), Duck calicivirus isolate NC-19BV-45-k77-747816 (‘Duck_NC-19BV’), grey teal calicivirus isolate MW09 ('Grey Teal'), Trumpeter swan calicivirus (‘Trumpeter’), MAG: Caliciviridae sp. isolate 361bR-k141_109560 (‘ Caliciviridae361bR’), Chicken calicivirus isolate Chicken/NLD/2019/V_M_036_calici_2 (‘Chicken_NLD_036’), MAG: Caliciviridae sp. isolate CAL/PB30-SII34/Switzerland/2019 (‘CAL/PB30-SII34’), Caliciviridae sp. isolate yc-13/Red-crowned crane calicivirus 1 ('RaCV1'), avocet calicivirus isolate MW21 ('Avocet'), Wilkes calicivirus isolate Antarctic11 ('Wilkes'), Pink-eared duck calicivirus I isolate MW23 ('Pink-eared duck'), Goose calicivirus isolate NC-19BV-37-k77-711556 (‘Goose_NC-19BV’), Caliciviridae sp. isolate xftoti59cal1 ('xftoti') and MAG: Duck calicivirus isolate PI202116/k141_160805 (‘Duck PI202116’). MAG: Caliciviridae sp. isolate CAL/PB27-SI31/Switzerland/2019 (GenBank: OM469262.1) and MAG: Caliciviridae sp. isolate CAL/PB10-HII34X/Switzerland/2019 (GenBank: OM469260.1) IRESs were excluded from analysis because they are 99% and 100% identical to MAG: Caliciviridae sp. isolate CAL/PB30-SII34/Switzerland/2019 (‘CAL/PB30-SII34’). Nucleotides that are identical in nine or more of the thirteen complete sequences are indicated in bold, and motifs containing these conserved nucleotides are shaded blue. Sequences were annotated after alignment to show boundaries of predicted domains and sequence motifs that are characteristic of type 2 IRESs.

Supplementary Figure S2. Aligned sequences of ORF1* sequences from *Caliciviridae* sp. isolate yc-13 (Red-crowned crane calicivirus 1) and related caliciviruses.

```
Avocet        --------------------MQTPTISDLRPIAQSGCILCVLDLQITLLATIKLQRWRLCQNHPN--  36
Turkey        -------------------------------------------------------------------   0
RaCV1         --------------------------------------------------------------MSNRP   5
Wilkes        ----------------------------------------------MPLPRQA--RHERSATTHNLS  19
Pink-eared    ----------MSMNAPPKSPLPHVATPHPLADPNVGLTISESSLTARMLKRRA-AR-DPTFSASTLT  55
Duck_PI2      ---------------MSTTTKRKSLPNPSQLQSPQNNQMSSHSLIALPLPRSQGG----SEQQCLIP  48
xftoti59Cal1  --------------------------------------MSSHSLIAHPLPRFQGG---SGQQCLIPP  26
DuCV2_B6      ------------------MSTKTQRKSSPNPSQSRMLQNNLTSLHLLTALHSGASRADSEQQCLNRP  49
Goose19BV     MTPTLKSTNNSSMPVSTSPRRNSQPAAFQLPSLSDLEKRPGSSLTARLLKRRRDRGF--ATPSSNHH  65
GooseN        -------------------------------------------------------------------   0
Duck2         -------------------------------------------------------------------   0

Avocet          -THLPLKHSAALRVLKLTLHTELPNVTTAGSAVLSSMAHASLKRTTNEPASHTHFLVQTPYRVCP 100
Turkey          -----------------MSSTTRDVLGTERGGASTPSPTVLQNAPIVQSVRPTRKKDLTPSTTSQ  48
RaCV1           PTPLPFRHSIVVNVPTPTLPTIGIVSNTDGDFTDTPYPVVLQRLITPDSAEPFPRMGVTHLMTCP  70
Wilkes          LLCVRSTPSTALTVPTSTRSTISAATSTGSISLPIPTQHALQQETMSRP--PTPYPMVTPSSSLR  82
Pink-eared      SICRLSKPSDATPALQSTPKQANTAKNTPNGTSPTSMPLASRRGITIEPVKLG---PRTPSQTSP 117
Duck_PI2        YTPPPSRRSAATRAQQPMQTTTGSADNTNGEDNLTFSPADSPNKITTESAKHSPSTDLTPSNLCP 114
xftoti59Cal1    STPSPSRRSAATRVQRHTQTITSSANNTTGEDNPTFSPADSLNKITLESARPSPSMDLTHSNSCP  90
DuCV2_B6        PTSTLSRPSAVTHVQQPMQTTTPSVGSTSGVLNPTYSPPDSPSKPTRQSVKQEPLMAQTPWSTSL 114
Goose19BV       PTCLPSTLSAVTHAQPHTSTTCSSAEDTSAEHSPISSPPALRRPTTLRLAAPSRPMDLTLWNQSP 130
GooseN          -------------------------------------------------------MNLIPWRKSP  10
Duck2           -----------------------------------------------------------------   0

Avocet          LMISGARFAPLRLTLPTVTSAQRSTLPPTIERALAMRNGAIQDPL-LRSAGTHQTVPSLATTSNCA 166
Turkey          LQSSGKPSQHLRRISHIATSAPSSTQVHTSGLASPGRNGLTSRRSVSRSENTHPPASLIPRVDLSY  94
RaCV1           PRPSGTPSDKWRKNTPNATSAQPFTPQLSTVLESPTLPGKTACLWRTNIESTPKS-ARLSKAVLRL 116
Wilkes          MQISGTRFAQSPKTQQSALFAPPLTAPPTTGVEPLSRSGSVTSLMSTSSVNTLKP-A-PSSHSLSS 128
Pink-eared      TPSSGPPSSTSSTNTLIASPVLPPTPQPTSEKAPLLKNGGNTSPVFFNFAGMPRR-VRSARTTLNY 182
Duck_PI2        HKNSGTRLSQSRKTLPSVASVRPPTPHFSTVLVDASMSGKPLSLNPTHSADTRRR-VRSARVNLNY 179
xftoti59Cal1    HKNSGTQSSQSRKTLPSVASVRPPTPHSSTVLVDVSTSGKPLSLNPTRSADTRRR-VRSARANLNY 137
DuCV2_B6        QGSSGTPSKPSRKTPPSATSVLPSTQPSSTEQEAASTSGKQHSPNPSHSADTRRR-VRSARANLNY 160
Goose19BV       HQSSGNLSEILKKSTPGATFVRRPTPPTSSEHPEDSTSGNEHSPARIASAATHPR-VRSARVNLNY 176
GooseN          RLNSGSLSEASKKSTPSATFVRRPTPPTSSEPLANLMTGKEPSPVPTTYAGTRRR-AR-AKVNLNY  65
Duck2           ------------------------------------------------------------------   0

Avocet        RAEDPHAWLDHQPRASTSAQ--PTPPLLADRQMRACSRQSPMP----------LAMRTSPPMGSFRRFR 213
Turkey        RAPETLRWLEPHLELECQDQ---------DSLQNSSVDHSCVPTLTSRGRYSPSLMEE--VMQ-PTLSP 145
RaCV1         REPDSPTR-EPPRVLEPPVKVTSSPPSVDLSLPLTVTSPHGSPDLLHLETTQ------------VTSSA 174
Wilkes        RALGIPLQ-EPFPELVPAGM---------LGVPACLQDPIKLRTTTSLPRSQQSLLTT-PPQP-HKSWM 176
Pink-eared    RAPETLPP-EPFPGLVPAAT---------LGLPASCQDPTKNRTLTSLHKSCQSSPPP-TPPL-LRYSS 239
Duck_PI2      RAPETLPP-APFPELERPTS---------LGLPMSCQDPTNVRTMISRQTSWDSLQT-NPPLP-RRSSL 236
xftoti59Cal1  RAPETLPP-VPFPELEHPTS---------LGLPMSCQDPTSVRTMISRQTSWDSLQTT-PPLP-RRSSL 186
DuCV2_B6      RAPETLPP-VPFPELEHPTS---------PGLPMSCQDPTSVRTMISRQTSWDSLQTT-PPLP-RRSSL 209
Goose19BV     RAPETLPP-EPFPELARLGS---------IGLPMSCLDPTKGRTRTSPPGSWASSPTT-PPPQ-PKSSR 225
GooseN        RAPETLPP-EPFPELAHLGS---------TGLPMNCRDPINDPTLISLRASWGSSPTT-PQPQ-PKLSR 105
Duck2         --------------------------------------------MISQQASWDSLQTT-PPLP-RRSSL  23

Avocet        KWLK--SKTQR----QHSHRHGDSLERIPLLTAP*---------------------------------- 239
Turkey        LLTPPLSRRQRSHLERLSVRSGDNY----TVTTAPIALQHYSLPSLSGTQTRMPWIRRIWRQFKQCVVK 236
RaCV1         RSTRRSTPTQTSLRVRPSLRSGATY----LPTTPPTELRPYLLLSQCGTETSTRSTDM-KLRCPGCVII 255
Wilkes        RSDPPLLLAPAPLRARPSHKYGAIS----ITTTRQTEQLRCSQQSPYGLQMQTRLTARNSRHCAPCTIL 268
Pink-eared    LWRTQSRTAPKRLPARPSRRFGETS----ATRMRQTEQLQYSLQSPSGSRTTTRSTVQSSIACAPCVVL 304
Duck_PI2      LSRLLSAQGPKLLPARPSRRFGAIS----TTTTRQTERLQSSLQSHSGLLTTTVSTQQNSRTCDPCVIQ 301
xftoti59Cal1  LSRLLFAQGPKLLPARPSRRFGAIS----TTTTRQTERLQSSLQSHSGLLTTTISTQQNSKTCDPCVIQ 278
DuCV2_B6      LSRLLSAQGPKLLPARPSRRFGAIS----TTTTRQTERLQSSLQSHSGLLTTTVSTQQNSKTCDPCVIQ 301
Goose19BV     LWRTLSERVPARLPARHSRRFGAIS----TMKTHQTEQLQFLLRSPSGSRITTRSTAQSSTTCDPCVIL 317
GooseN        LWRIPSSRVPARLPARPSRKFGAIS----TTRMHQTEQLQYSQLSPSGSRTTTRSTVQNSTTCDPCVIL 197
Duck2         LSRLLSAQGPKLLPARPSRRFGAIS----TTTTRQTERLQSSLQSHSGLLTTTVSTQQNSKTCDPCVIQ  88
```

Supplementary Figure S2. Aligned sequences of ORF1* sequences from avocet calicivirus isolate MW21 ('Avocet'), Turkey calicivirus isolate L11043 ('Turkey'), Caliciviridae sp. isolate yc-13/Red-crowned crane calicivirus 1 ('RaCV1'), Wilkes calicivirus isolate Antarctic11 ('Wilkes'), Pink-eared duck calicivirus I isolate MW23 ('Pink-eared'), MAG: Duck calicivirus isolate PI202116/k141_160805 ('Duck_PI2'), Caliciviridae sp. isolate xftoti59cal1 ('xftoti59Cal1'), Duck calicivirus 2 strain DuCV-2_B6, Goose isolate NC-19BV-37-k77-711556 ('Goose19BV'), Goose calicivirus strain N ('GooseN') and Duck calicivirus 2 strain DuCV-2_B76 ('Duck2'). Amino acid residues conserved in 70% or more of sequences are in bold font and highlighted blue.

Supplementary Figure S3. Aligned sequences of ORF1* sequences from Caliciviridae sp. isolate CAL/PB27-SI31/Switzerland/2019 and related caliciviruses.

```
MZ679042.1          METWTLVQKTTSISLLPLPFKAKHSYRTLSASVRPSTNLPASSVPKTFARLQSTAGHINEAFQDTDTMPD   70
CAL/PB27-SI31       METWTLVQKTTSISLLPLPFKAKHSYRTLSASVRPSTNLPVSSVPKTFARLQSTAGHINEAFQDTDTMPD   70
CAL/PB30-SII34      METWTLVQKTTSISLLPLPFKAKHSYRTLSASVRPSTNLPASSVPKTFARLQSTAGHINEAFQDTDTMPD   70
CAL/PB10-HII34X     METWTLVQKTTSISLLPLPFKAKHSYRTLSASVRPSTNLPASSVPKTFARLQSTAGHINEAFQDTDTMPD   70
MW684834.1          MESWTLVQKTTSISLLPLPFKAKHSYRTLSASVRPSTNSPASSVPKTFARLQSTVGHINEAFQDTDTMPD   70
MW684835.1          MESWTLVQKTTSISLLPLPFKAKHSYRTLSASVRPSTNSPASSVPKTFARLQSTVGHINEAFQDTDTMPD   70
MW684841.1          MESWTLVQKTTSISLLPLPFKAKHSYRTLSASVRPSTNSPASSVPKTFARLQSTVGHINEAFQDTDTMPD   70
MW684842.1          MESWTLVQKTTSISLLPLPFKAKHSYRTLSASVRPSTNSPASSVPKTFARLQSTVGHINEAFQDTDTMPD   70
MW684843.1          MESWTLVQKTTSISLLPLPFKAKHSYRTLSASVRPSTNSPASSVPKTFARLQSTVGHINEAFQDTDTMPD   70
MW684844.1          MESWTLVQKTTSISLLPLPFKAKHSYRTLSASVRPSTNSPASSVPKTFARLQSTVGHINEAFQDTDTMPD   70
MW684840.1          MESWTLVQKTTSISLLPLPFKAKHSYRTLSASVRPSTSLPASSVPKTFARLQSTVGHINEAFQDTDTMPD   70
MW684838.1          MESWTLVQKTTSISLLPLPFKAKHSYRTLSASVRPSTNSPASSVPKTFARLQSTVGHIN-AFQDTDTMPD   69
MW684845.1          MESWTLVQKTTSISLLPLPFKAKHSYRTLSASVRPSTNSPASSVPKTFARLQSTAGHINEAFQDTDTMPD   70
MW684846.1          ----------------------------------------------------------------------   0
MW684847.1          MESWTLVQKTTSISLLPLPFKAKHSYRTLSASVRPSTNSPASSVPKTFARLQSTAGHINEAFQDTDTMPD   70

MZ679042.1          SPDCPTACNAVAMEPSLNSSTNPSASNMKSTGSRNIPTASSVPFSSQLLSLSEEYLSQLGNAPCPSASAT   140
CAL/PB27-SI31       SPDCPTACNAVVMEPSLNSSTSLSASNMKSTESRNTPTASSVPFSSQLLSLSEEYLSQLGSAPCPSASAT   140
CAL/PB30-SII34      SPDCPTACNAVAMEPSLNSSTSLSASNMKSTESRNTPTASSVPFSSQLLSLSEEYLSQLGSAPCPSASAT   140
CAL/PB10-HII34X     SPDCPTACNAVAMEPSLNSSTSLSASNMKSTESRNTPTASSVPFSSQLLSLSEEYLSQLGSAPCPSASAT   140
MW684834.1          SPDCPTACNAAAMVPSQNSSTSQSASNTKLIVSRNTPTASSVPFSSQLLSLSEEYLSQLGNAPCPSASAT   140
MW684835.1          SPDCPTACNAAAMVPSQNSSTSQSASNTKLIVSRNTPTASSVPFSSQLLSLSEEYLSQLGNAPCPSASAT   140
MW684841.1          SPDCPTACNAAAMVPSQNSSTSQSASNTKLIVSRNTPTASSVPFSSQLLSLSEEYLSQLGNAPCPSASAT   140
MW684842.1          SPDCPTACNAAAMVPSQNSSTSQSASNTKLIVSRNTPTASSVPFSSQLLSLSEEYLSQLGNAPCPSASAT   140
MW684843.1          SPDCPTACNAAAMVPSQNSSTSQSASNTKLIVSRNTPTASSVPFSSQLLSLSEEYLSQLGNAPCPSASAT   140
MW684844.1          SPDCPTACNAAAMVPSQNSSTSQSASNTKLIVSRNTPTASSVPFSSQLLSLSEEYLSQLGNAPCPSASAT   140
MW684840.1          SPDCPTACNAAAMVPSQSLSTSQSASSTKLTVSRSTPTASSVPFSSQLLSLSEEYLSQLGNAPCPSASAT   140
MW684838.1          SPDCPTACNAVAMVPSQNSSTNQSASSTKLTVSRSTPIASSVPFSSQLLSLSEEYLSQLGNAPCPSASAT   139
MW684845.1          SPDCPTACNAAAMVPSQNSSTSQSASSTKLTVSGNTPTASSVPFSSQLLSLSEEYLSQLGNAPCPSASAT   140
MW684846.1          ----------------------------------------------------------------------   0
MW684847.1          SPDCPTACNAAAMVPSQNSSTSQSASSTKLTVSGNTPTASSVPFSSQLLSLSEEYLSQLGNAPCPSASAT   140

MZ679042.1          IAGTPKSAALSQPSLPPRRWKLLRHRNLWRRSSRPMVQTPPLSTPPATTQLDGPETRL*      198
CAL/PB27-SI31       IADTPKSAALSQPSLPPRRWKLLRHRNLWRRSSRPTVQTPPLSTPPATTQLDGPETRL*      198
CAL/PB30-SII34      IADTPKSAVLSQPSLPPRRWKLLRHRNLWRRSSRPTVQTPPLSTPPATTQLDGPETRL*      198
CAL/PB10-HII34X     IADTPKSAALSQPSLPPRRWKLLRHRNLWRRSSRPTVQTPPLSTPPATTQLDGPETRL*      198
MW684834.1          IADTPKSAALSQPSLPPRRWKFLRHRNLWRRSSRPTVQTPLLSTPPAITQLDGLETRL*      198
MW684835.1          IADTPKSAALSQPSLPPRRWKFLRHRNLWRRSSRPTVQTPLLSTPPAITQLDGLETRL*      198
MW684841.1          IADTPKSAALSQPSLPPRRWKFLRHRNLWRRSSRPTVQTPLLSTPPAITQLDGLETRL*      198
MW684842.1          IADTPKSAALSQPSLPPRRWKFLRHRNLWRRSSRPTVQTPLLSTPPAITQLDGLETRL*      198
MW684843.1          IADTPKSAALSQPSLPPRRWKFLRHRNLWRRSSRPTVQTPLLSTPPAITQLDGLETRL*      198
MW684844.1          IADTPKSAALSQPSLPPRRWKFLRHRNLWRRSSRPTVQTPLLSTPPAITQLDGLETRL*      198
MW684840.1          IADTPKSAALSQPSLPPRRWKLLRHRNLWRRSSRPTVQTPLLSTPPATTQLDGLETKL*      198
MW684838.1          IADTPKSAALSQPSLPPRRWKLLRHRNLWRRSSRPTVQTPLLSTPPATTQLDGLETKL*      197
MW684845.1          IADTPKSAALSQPSLPPRRWKLLRHRNLWRRSSRPTVQTPLLSTPPATTQLDGPETKL*      198
MW684846.1          -ADTPKSAALSQPSLPPRRWKLLRHRNLWRRSSRPTVQTPLLSTPPATTQLDGPETKL*      57
MW684847.1          IADTPKSAALSQPSLPPRRWKLLRHRNLWRRSSRPTVQTPLLSTPPATTQLDGPETKL*      198
```

Supplementary Figure S3. Aligned sequences of ORF1* sequences from MAG: Caliciviridae sp. isolat e 361bR-k141_109560 (GenBank: MZ679042.1), MAG: Caliciviridae sp. isolate CAL/PB27-SI31/Switzerland/2019 (GenBank: OM469262.1), MAG: Caliciviridae sp. isolate CAL/PB30-SII34/Switzerland/2019 (GenBank: OM469263.1) MAG: Caliciviridae sp. isolate CAL/PB10-HII34X/Switzerland/2019 (GenBank: OM469260.1), MAG: Chicken calicivirus isolate Environment/NLD/2019/VE_8_calici_66 (GenBank: MW684834.1), MAG: Chicken calicivirus isolate

Chicken/NLD/2019/V_M_019_calici_3 (GenBank: MW684835.1), MAG: Chicken calicivirus isolate Chicken/NLD/2019/V_M_041_calici_11 (GenBank: MW684841.1), MAG: Chicken calicivirus isolate Chicken/NLD/2019/V_M_050_calici_2 (GenBank: MW684842.1), MAG: Chicken calicivirus isolate Chicken/NLD/2019/V_M_051_calici_5 (GenBank: MW684843.1), MAG: Chicken calicivirus isolate Chicken/NLD/2019/V_M_052_calici_7 (GenBank: MW684844.1), MAG: Chicken calicivirus isolate Chicken/NLD/2019/V_M_036_calici_2 (GenBank: MW684840.1), MAG: Chicken calicivirus isolate Chicken/NLD/2019/V_M_030_calici_8 (GenBank: MW684838.1), MAG: Chicken calicivirus isolate Chicken/NLD/2019/V_M_053_calici_1 (GenBank: MW684845.1), MAG: Chicken calicivirus isolate Chicken/NLD/2019/V_M_054_calici_1 (GenBank: MW684846.1) and MAG: Chicken calicivirus isolate Chicken/NLD/2019/V_M_056_calici_1 (GenBank: MW684847.1). Amino acid residues that differ from the consensus are in bold font and highlighted blue.

Supplementary Figure S4.

A) Aligned ORF1* sequences from *Caliciviridae* sp. isolate cftwhg06cal1 (nt. 599-1243) and Goose calicivirus isolates MA/CHN/2018 (nt. 77-517) and H146 (nt. 80-520). The symbols "*", ":" and "." indicate identical amino acid residues, conservative substitutions and semi-conservative substitutions, respectively. Numbering refers to amino acid residues.

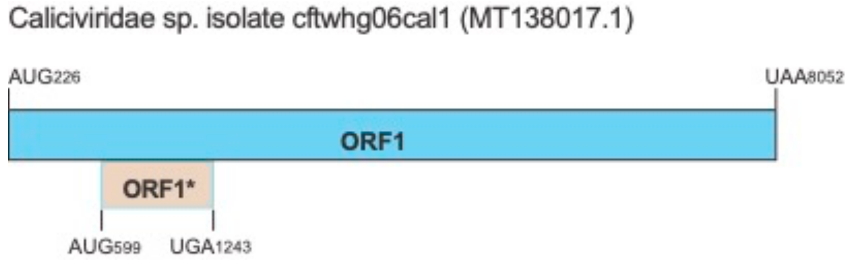


```
cftwhg06cal1      MHPMDASFLTRPTPLASALSGQIPMLTQILSGRIAKGKSSGLHVLLKTLRRVVLCSKIHL    60
MA/CHN/2018       ------------------------------------------------------------    0
H146              ------------------------------------------------------------    0

cftwhg06cal1      TRNERLCTSTTVRRWLNGSRKELSTRSAWQFLHLSSEKRVFKFHTLTLMLPLLKLRMPGK    120
MA/CHN/2018       -------MSTTVRRWLNGSKRELSTRSAWLYLHLSSEKRVFKFHTLTLMLPLRTLRMPGK    53
H146              -------MSTTVRRWLNGSKRELSTKSAWLYLHLSSEKRVFKFHTLTLMLPLRTLRMPGK    53
                         ***********::****:*** :********************* .******

cftwhg06cal1      SSSFVGSLSTILAKLSFMARPLTRLRVVRFRFRDRFLLMRRTRMFLTITLLRVMVQRMSN    180
MA/CHN/2018       SSSFARSLSTTWAKQNCMDPPLIKSRVVKFRCRDQFLPTKRTRMFLITTPLKGMGQRTSN    113
H146              SSSFARSLSTTWAKQNCMDPPLIKLRVVKFRYRDRFLLTKKTRMSLITTPLRGMGQRTSN    113
                  ****. ****  ** . *  ** : ***:** **:**  ::*** *  * *: * ** **

cftwhg06cal1      SSMNGLHPLMPSKTGLKQCPLLQMLCQMLILTQS*                             214
MA/CHN/2018       SFMNGSPPLSLSNNGSKQCPLPRTMCQMLHLIQN*                             147
H146              SFTNGSPPLSPSNNGSKQCPLPRTLCQMLHLIQN*                             147
                  *  **  **  *:.* ***** : :**** * *.
```

B) Aligned ORF1* sequences from Duck calicivirus isolate sMW20 (nt. 883-1339) and NC-19BV-45-k77-747816 (nt. 895-1317). The symbols "*", ":" and "." indicate identical amino acid residues, conservative substitutions and semi-conservative substitutions, respectively. Numbering refers to amino acid residues.

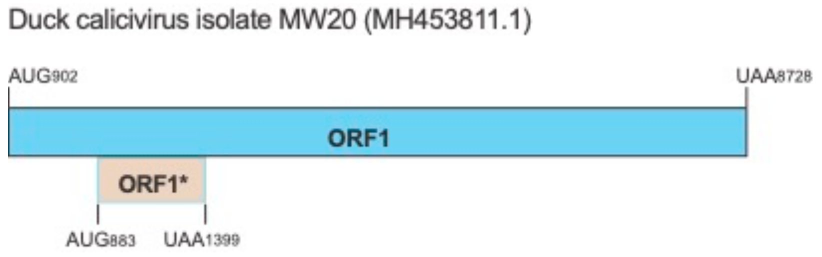


```
DuckMW20          MVQIEYHGLITLSTRAVSHYYGGFFVSMLEVFRPRLLSLNIHFGIADGAPFLFYQESRKC    60
Duck_NC-19BV      --MIFYHGVIQTTAWTIEQVLGSLPLRILNYGLPPLLSYTIRFGWGTSGPFFWVQESRKC    58
                     * ***:*  :: ::.:  *.: : :*:   * *** .*:** . ..**:: ******

DuckMW20          PPGGRCRCCVRYLVGHVRIG-HLVGREPDFFAPL-----NEWYVEERVAQLLPAVGEGEE    114
Duck_NC-19BV      PASGPCQCRLHRLVGESYTTRDIFAREPDRTIELFLSGIEEEEVRERLD--LRPLAEGSE    116
                  * .* *:* :: ***.     .:..****    *     :*  *.**:   *  :.**.*

DuckMW20          GTEGDRGTPVKDRQEDSRHLRHRAVRGDCGRPGPNDVF*                         152
Duck_NC-19BV      G--------------RARYGGHSSVRGRGRGPGINDAF*                         140
                  *               :*:  * :***    ** **.*
```

C) ORF1* sequence from MAG: Caliciviridae sp. isolate ybb044cal01nc (nt. 205-702). Numbering refers to amino acid residues.

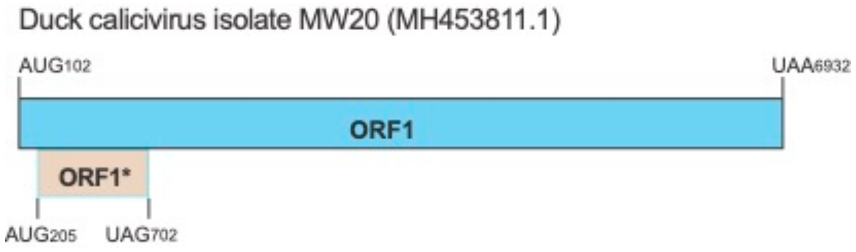


```
MTPPPSRFLTPLRARLAATCGENWTWTAPLSRGWLPYTRLSTARGATCYGRVPRAQTTYL         60
KSDSLTLPLTPRHDPPPSASACGPSCTGSLTTCCSTLCKLDTPPTLPGPCSHYCGPQLCG         120
ASCQRSKAGTSLVSGLSLSFTVWRTPLTPPMWRASLSSFRKSCQP*                       165
```

Supplementary Figure S5

Open reading frames (ORFs) upstream of, overlapping or fused to ORF1 in chicken calicivirus genomes

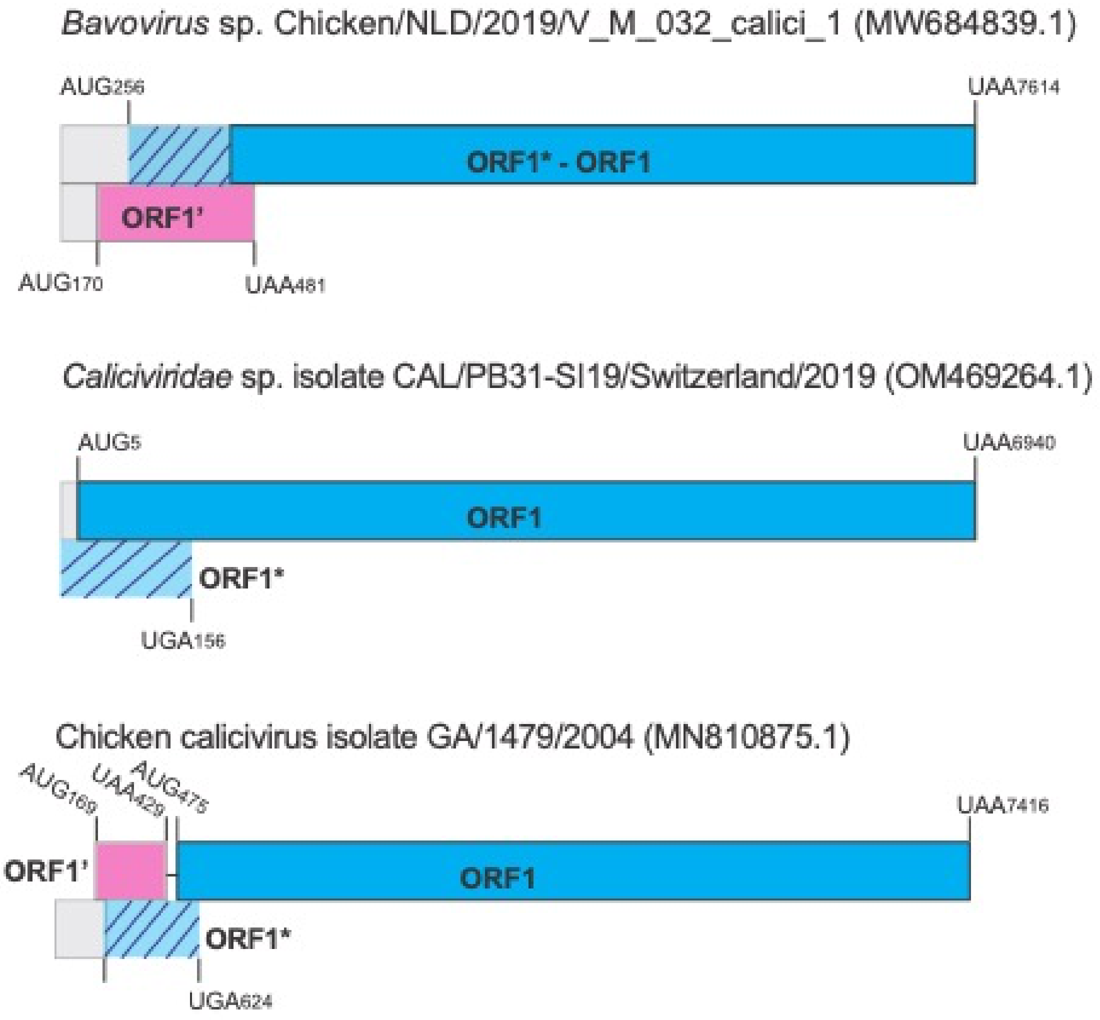

A) Schematic representations of the 5'-termini and ORF1 of *Bavovirus* sp. Chicken/NLD/2019/V_M_032_calici_1, *Caliciviridae* sp. isolate CAL/PB31-SI19/Switzerland/2019 (OM469264.1) and Chicken calicivirus isolate GA/1479/2004, showing ORF1 (cyan) and its annotated initiation and termination codons, ORF1* (striped), ORF1' (magenta) and unannotated potential N-terminal extensions of ORF1, ORF1* and ORF1' (light grey). ORF1* and ORF1 are fused in *Bavovirus* sp. Chicken/NLD/2019/V_M_032_calici_1.

B) Alignment of calicivirus ORF1’ sequences

```
Chicken/NLD/2019/V_M_032_calici_1    2                                                 RKGFTKLTPVGAAVGKEARASI  67


Chicken calicivirus GA/1479/2004     7    TLSSNFGHGPLSVFPSLKRASAGASQLGPQKSPGWNSTVTGVTLTVIQPPSLLVMGASSQ  186
Chicken calicivirus GA/1478/2003     7    TLSSNFGHGLLSVFPSSKRASAGVSQLRPQKSPGWNSMVTGVTLTVIQPPSLLVMGASSQ  186
Chicken/NLD/2019/V_M_032_calici_1   68       TNVGHGLPSVLPSLKRASAGVSQLGPQKSPGWNSMVIGVTSTVTQPPSLPVMGASSL  238
Chicken calicivirus [ChCV-052]       1  VGTLSSNSGHGLLSVFPSSKRASAGASRLGPQESPGWISMATGVALTVIPPHSLLVKGASSL  186
Chicken calicivirus RS/BR/15/1R-1    5          SGHGPLSVFPSSKRASAGVSRLGPQESPGWNSMATGVALTVIPPHSLLVKGASSL  166
Chicken calicivirus RS/BR/15/4R-1    3                                              VTLTVIPPHSLLVMGASSL  59
Chicken calicivirus RS/BR/15/4R-1    8                                           AIGVALTVIPPHSLLVKGASSL  67
Chicken calicivirus RS/BR/15/3S-1    1                                                         LVKGASSL  24
Chicken calicivirus RS/BR/15/6R      2                                                         LVMGASSL  25
Chicken calicivirus RS/BR/2015       1                                                            GASSL  15


Chicken calicivirus GA/1479/2004   187    NTLEPRCRVGSHSCPLATCRFLTPSSAPTSSPGWCNTPRLMNRLGGTSGQPGPSRTSGEF  366
Chicken calicivirus GA/1478/2003   187    NTPDLRCRVGSHSCPLATCRYPTPSSAPTFSPGWYSTPRLMNRPGRTSGQPGPSRISGES  366
Chicken/NLD/2019/V_M_032_calici_1  239    NTLGLRCRVGSRSCPLATCRCLTPNSALTSFPGWYNTPRLMNKPGGISGQPGPSRISAGS  418
Chicken calicivirus [ChCV-052]     187    NTLGQRCKGGSRSCPLATCRCPIPSSALTSSPGWCNTPRLMNKPGGTSRQPGLSRISGAS  366
Chicken calicivirus RS/BR/15/1R-1  167    NTLGQRCKGGSHSCPLATCRCPIPSLVLTSFPGWCNTPRLMNKPGGTSGQPGLSRISGAS  346
Chicken calicivirus RS/BR/15/4R-1   60    NTLGQRCKGGNHSCPLATCRYPTPSLALTSSPGWCNTLRLMNKPGGTSGQPGLSRISGAF  239
Chicken calicivirus RS/BR/15/4S-1   68    NTLGQRCKGGSHSCPLATCRCPIPSLALTSFPGWCNTPRLMNKPGGTSGQPGLSRISGAS  247
Chicken calicivirus RS/BR/15/3S-1   25    NTLGQRCKGGSHSCPLATCRCPTPSLVLTFSPGWCNTPRLMNKPGGTSGQPGLSRISGAS  204
Chicken calicivirus RS/BR/15/6R     26    NTLGQRCKGGNHSCPLATCRYPTPSLALTSSPGWYNTLRLMNKPGGTSGQPGLLRISGAS  205
Chicken calicivirus RS/BR/2015      16    NTLGQRCKGGSHSCPLATCRCPIPSLVLTSSPGWCNTPRLMNKPGGTSGQPGLSRISGAS  195
Bavaria virus A5_2S5_SA_2022         2          RSRVGSRSCLLATCRCLTPSSAPTSSPG*CNTPRLMNRPGGTSGQPGPLRIFGES  166
Chicken/NLD/2019/V_M_021_calici_5.   1                                                          SRISAGS   21


Chicken calicivirus GA/1479/2004   367    LLTTSCSQNLRKTLMPMMAG*   426
Chicken calicivirus GA/1478/2003   367    LPTTSCSQNLRRTLMPMMAG*   426
Chicken/NLD/2019/V_M_032_calici_1  419    PLTTSCSQNLRKTLMPMMAG*   478
Chicken calicivirus [ChCV-052]     367    PQTTSCSRNLRRTLMPMMAG*   426
Chicken calicivirus RS/BR/15/1R-1  347    PQTTSCSRNLRRTLMPMMAG*   406
Chicken calicivirus RS/BR/15/4R-1  240    LQTTSCSRNLKRILMPMMAG*   299
Chicken calicivirus RS/BR/15/4S-1  248    PQTTSCSRNLRRTLMPMMAG*   307
Chicken calicivirus RS/BR/15/3S-1  205    PQTTSCSRNLRRTLMPMMAG*   264
Chicken calicivirus RS/BR/15/6R    206    LQTTSCSRNLRRILMPMMAG    265
Chicken calicivirus RS/BR/2015     196    PQTTSCSRNLRRTLMPMMAG*   255
Bavaria virus A5_2S5_SA_2022       167    LPTTSCSQNLKRTLMPMMVG*   226
Chicken/NLD/2019/V_M_021_calici_5   22    PLTTSCSQNLRKTLMPMMAG*    85
```

Sequence alignment of ORF1’ sequences from Chicken calicivirus isolate GA/1479/2004 (MN810875.1), Chicken calicivirus isolate GA/1478/2003 (MN810874.1), *Bavovirus* sp. Chicken/NLD/2019/V_M_032_calici_1 (MW684839.1), Chicken calicivirus [ChCV-052] (MH992118.1), Chicken calicivirus strain RS/BR/15/1R-1 (MG846434.1), Chicken calicivirus strain RS/BR/15/4R-1 (MG846428.1), Chicken calicivirus strain RS/BR/15/4S-1 (MG846430.1), Chicken calicivirus strain RS/BR/15/3S-1 (MG846429.1), Chicken calicivirus strain RS/BR/15/6R (MG846433.1), Chicken calicivirus strain RS/BR/2015 (NC_033081.1), Bavaria virus isolate A5_2S5_SA_2022 (PP228889.1) and *Bavovirus* sp. isolate Chicken/NLD/2019/V_M_021_calici_5 (MW684836.1). Numbering refers to nucleotides in genmic sequences.

ORF1’ is located upstream of ORF1 and does not overlap it, except in Bavaria virus isolate A5_2S5_SA_2022 and *Bavovirus* sp. Chicken/NLD/2019/V_M_032_calici_1, in which the ORF1’ sequence overlaps ORF1 entirely, and in the former, is interrupted by a stop codon. Potential AUG initiation codons within ORF1’ are in bold and highlighted blue.

C) Alignment of calicivirus ORF1* sequences

```
Chicken/NLD/2019/V_M_032_calici_1    1                                     EERVHKADPRRGSSGKGGKSLHN  69


Chicken calicivirus ChCV-052         3 GNPFLKLRTRTTLRVSLIKEGLSWRKSAGAPRITWVDLYGDWGRFDCDPTPLFAREGCIL 182
Chicken calicivirus RS/BR/15/1R-1    1       LRTRTTLRVSLIKEGLSWRKSAGAPRITWVELYGDWGRFDCDPTPLFAREGCII 162
Chicken calicivirus GA/1479/2004     3 VNPFLKLRTRTTLRVSLIKEGLSWRKSAGAPKISWVELYGDWGHFDCDPTPLFARDGCII 182
Chicken calicivirus GA/1478/2003     3 VNPFLKLRTRTTLRVSLIKEGLSWRKSAEAPKISWVELYGDWGHFDCDPTPLFARDGCII 182
Chicken/NLD/2019/V_M_032_calici_1   70      KCRTRTTLRASLIKEGLSWRKPTGAPKISWVELYGDWGHFDCDPTPLFARDGCIV 234
Chicken calicivirus RS/BR/15/4S-1    1                                        GDWGRFDCDPTPLFAREGCII  63
Chicken calicivirus RS/BR/15/4R-1    2                                           GHFDCDPTPLFARDGCII  55
Chicken calicivirus RS/BR/15/3S-1    3                                                       REGCII  20
Chicken calicivirus RS/BR/15/6R      1                                                      ARDGCII  21
Chicken calicivirus RS/BR/2015       3                                                          CII  11


                                               #
Chicken calicivirus ChCV-052       183 TEHPRAEMQGWKSLLPPGNMQVPDPKLSSDFLSWVVQHSKVDEQTWRDLKATWSLEDLRR 362
Chicken calicivirus RS/BR/15/1R-1  163 TEHPRAEMQGWKSLLPPGNMQVPDPKLSSDFLSWVVQHSKVDEQTWRDLWATWSLEDLRR 342
Chicken calicivirus GA/1479/2004   183 TEHPRAEMQGWKSLLPPGNMQVPDPKLSSDFLSWVVQHSKVDEQTWRDLWATWSLEDLRR 362
Chicken calicivirus GA/1478/2003   183 TEHPRSEMQGWKSLLPPGNMQVPDPKLSSDFLSWVVQHSKVDEQTWKDLWATWSLEDLRR 362
Chicken/NLD/2019/V_M_032_calici_1  235 TEHPRAEMQGWKSLLPPGNMQVPDPKLSSDFLSWVVQHSKVDEQTWRDLWATWSLEDLRR 414
Chicken calicivirus RS/BR/15/4S-1   64 TEHPRAEMQGWKSLLPPGNMQVPDPKLSSDFLSWVVQHSKVDEQTWRDLWATWSLEDLRR 243
Chicken calicivirus RS/BR/15/4R-1   56 TEHPRAEMQGWKSLLPPGNMQVPDPKLSSDFLSWVVQHSKVDEQTWRDLWATWSLEDLRR 235
Chicken calicivirus RS/BR/15/3S-1   21 TEHPRAEMQGWKSLLPPGNMQVPDPKLSSNFLSWVVQHSKVDEQTWRDLWATWSLEDLRR 200
Chicken calicivirus RS/BR/15/6R     22 TEHPRAEMQGWKSLLPPGNMQVPDPKLSSDFLSWVVQHSKVDEQTWRDLWATWSLEDLRR 201
Chicken calicivirus RS/BR/2015      12 TEHPRAEMQGWKSLLPPGNMQVPDPKLSSDFLSWVVQHSKVDEQTWRDLWATWSLEDLRR 191
Bavaria virus A5_2S5_SA_20221        3       EIQGWKSLLPPGNMQVPDPKLSSDFLSWVVQHSKVDEQTWRDLWATWSLEDLRR 162
Chicken calicivirus CaliciD62/2013   1                                                       LEDLRR  18
Chicken/NLD/2019/V_M_021_calici_5    3                                                        EDLRR  17


Chicken calicivirus [ChCV-052]     363 ISTDYLMLPEPEKNTDAYDGWLMVGEMLTLAGLIGWEDDDGDLRFGSNDTYWGLFGDPATVRK 551
Chicken calicivirus RS/BR/15/1R-1  343 ISTDYLMLPEPEKNTDAYDGWLMVGEMLTFAGLIGWEDDDGDLSFGSNHTYWGLFGDPATVRK 531
Chicken calicivirus GA/1479/2004   363 ISTDYLMLPEPEKNTDAYDGWLMVGEMLTFAGLIGWEDDDGDLSFGSNHTYWGLFGDPATVRK 551
Chicken calicivirus GA/1478/2003   363 ISTDYLMLPEPEKNTDAYDGWLMVGEMLTFAGLIGWEDDDGDLSFGSNHTYWGLFGDPATVRK 551
Chicken/NLD/2019/V_M_032_calici_1  415 ISTDYLMLPEPEKNTDAYDGWLMVGEMLTFAGLIGWEDDDGDLSFGSNHTYWGLFGDPATVRK 603
Chicken calicivirus RS/BR/15/4S-1  244 ISTDYLMLPEPEKNTDAYDGWLMVGEMLTFAGLIGWEDDDGDLSFGSNHTYWGLFGDPATVRK 432
Chicken calicivirus RS/BR/15/4R-1  236 ISTDYLMLPEPEKNTDAYDGWLMVGEMLTFAGLIGWEDDDGDLSFGSNHTYWGLFGDPATVRK 424
Chicken calicivirus RS/BR/15/3S-1  201 ISTDYLMLPEPEKNTDAYDGWLMVGEMLTFAGLIGWEDDDGDLSFGSNHTYWGLFGDPATVRK 389
Chicken calicivirus RS/BR/15/6R    202 ISTDYLMLPEPEKNTDAYDGWLMVGEMLTFAGLIGWEDDDGDLSFGSNHTYWGLFGDPATVRK 390
Chicken calicivirus RS/BR/2015     192 ISTDYLMLPEPEKNTDAYDGWLMVGEMLTFAGLIGWEDDDGDLSFGSNHTYWGLFGDPATVRK 380
Bavaria virus A5_2S5_SA_2022       163 ISTDYLMLPEPEKNTDAYDGWLMVGEMLTFAGLIGWEDDDGDLSFGSNHTYWGLFGDPATVRK 351
Chicken calicivirus CaliciD62/2013  19 ISTDYLMLPEPEKDTDAHDGWLMVGEMLTFAGLIGWEDDDGELSFGSNHTYWGLFGDPATVRK 207
Chicken/NLD/2019/V_M_021_calici_5   18 ISTDYLMLPEPEKNTDAYDGWLMVGEMLTFAGLIGWEDDDGDLSFGSNHTYWGLFGDPATVRK 206
Chicken/NLD/2019/V_M_029_calici_1    3         EPEKNTDAYDGWLMVGEMLTFAGLIGWEDDDGDLSFGSNHTYWGLFGDPATVRK 164
CAL/PB31-SI19/Switzerland/2019       1                                EDDDGDLSFGSNHTYWGLFGDPATVRK  81
Calici chicken/V0021/Bayern/2004     4                                 DEDGDLSFGSNHTYWGLFGDPATVRK  81
```

```
Chicken calicivirus [ChCV-052]     552 KVDGLFHDIRTGGSRHPSNHHL*     620
Chicken calicivirus RS/BR/15/1R-1  532 KVDGLFHDIRTGGSRHPSNHHL*     600
Chicken calicivirus GA/1479/2004   522 KVDGLFHDIRTGDSRPPGNHHAHT*   626
Chicken calicivirus GA/1478/2003   552 KVDGLFHDIRTGDSRPPGNHHAHT*   626
Chicken/NLD/2019/V_M_032_calici_1  604 KVDGLFHDIRTGGSCPQATTSPTPEPNPTPEQVIPNPPEPSFWRRLIDGVTPPQEPPLLSAITS...
Chicken calicivirus RS/BR/15/4S-1  433 KVDGLFHDIRTGGSRHPSNHHA*     501
Chicken calicivirus RS/BR/15/4R-1  425 KVDGLFHDIRTGGSRHPSNHHS*     493
Chicken calicivirus RS/BR/15/3S-1  390 KVDGLFHDIRTGGSRHPSNHHL*     458
Chicken calicivirus RS/BR/15/6R    391 KVDGLFHDIRTGGSRHPSNHHS*     459
Chicken calicivirus RS/BR/2015     381 KVDGLFHDIRTGGSRHPSNHHL*     449
Bavaria virus A5_2S5_SA_2022       352 KVDGLFHDIRTGGSSTQAPTAPTPEPTSTSEQVLPNPPEPSFWRRLIDGVTPPQEPPLLSAITS...
Chicken calicivirus CaliciD62/2013 208 KVDGLFHDIRTGGSRPPSNHHAHT*   282
Chicken/NLD/2019/V_M_021_calici_5  207 KVDGLFHDIRTGGSCPQATTSPTPEPNPTPEQVIPNPPEPSFWRRLIDGVTPPQEPPLLSAITS...
Chicken/NLD/2019/V_M_029_calici_1  165 KVDGLFHDIRTGGSCPQATTSPTPEPNPTPEQVIPNPPEPSFWRRLIDGVTPPQEPPLLSAITS...
CAL/PB31-SI19/Switzerland/2019      82 KVDGLFHDIRTGSSRAPSLCTARL*   156
Calici chicken/V0021/Bayern/2004    82 KVDGLFHDIRTGSSRPPSNYNARS*   156
```

ORF1 sequences

```
Chicken calicivirus [ChCV-052]     598                  TQATTTSSPEVNTNSEQVLPNPPEPSFWHRLIDGVTPPPEPPHLSANTS 744
Calici chicken/V0021/Bayern/2004   128                  PQATTTPAPETNPTSEQVLPNPPEPSFWHRLIDGVAPPQEPPLLSAITS 274
CAL/PB31-SI19/Switzerland/2019     128                  PQASAPPASETNSTSEQVLPNPPEPSFWHRLIDGVAPPQEPPLLSAITS 274
Chicken_calicivirus_CaliciD62/2013 255                  PQATTTPTPEATSSSEQVLPNPPEPSFWHRLIDGVAPPQEPPLLSAITS 400
Chicken_calicivirus_GA/1478/2003   597                  PQATTMPTPEANTASEQVLPNPPEPSFWRRLIDGVTPPQEPPLLSAITS 744
Chicken_calicivirus_GA/1479/2004   597                  PQATTMPTPEANTNSEQVLPNPPEPSFWRRLIDGVTPPQEPPLLSAITS 744
Chicken_calicivirus_RS/BR/15/3S-1  460         ---------------PEANTNSEQVLPNPPEPSFWHRLIDGVTPPQEPPLLSAITS 582
Chicken-calicivirus_RS/BR/15/4S-1  503         ---------------PEASTNTEQVLPNPPEPSFWHRLIDGVTPPQEPPLLSAITS 625
Chicken-calicivirus_RS/BR/15/4R-1  494         ---------------PEASTNTEQVLPTPPEPSFWLRLIDGVTPPQEPPLLSAITS 616
Chicken_calicivirus_RS/BR/15/6R    461         ---------------PEASTNTEQVLPNPPEPSFWHRLIDGVTPPQEPPLLSAITS 583
Chicken_calicivirus_RS/BR/15/1R-1  602         ---------------PEANTNSEQVLPNPPEPSFWHRLIDGVTPPQEPPLLSAITS 724
Chicken_calicivirus_RS/BR/2015     451         ---------------PEANTNSEQVLPNPPEPSFWHRLIDGVTPPQEPPLLSAITS 573
```

Sequence alignment of ORF1* and ORF1 sequences from Chicken calicivirus strain ChCV-052 (MH992118.1), Chicken calicivirus strain RS/BR/15/1R-1 (MG846434.1), Chicken calicivirus isolate GA/1479/2004 (MN810875.1), Chicken calicivirus isolate GA/1478/2003 (MN810874.1), *Bavovirus* sp. Chicken/NLD/2019/V_M_032_calici_1 (MW684839.1), Chicken calicivirus strain RS/BR/15/4S-1 (MG846430.1), Chicken calicivirus strain RS/BR/15/4R-1 (MG846428.1), Chicken calicivirus strain RS/BR/15/3S-1 (MG846429.1), Chicken calicivirus strain RS/BR/15/6R (MG846433.1), Chicken calicivirus strain RS/BR/2015 (NC_033081.1), Bavaria virus isolate A5_2S5_SA_2022 (PP228889.1), Chicken calicivirus isolate CaliciD62/2013 (KM254170.1), *Bavovirus* sp. isolate Chicken/NLD/2019/V_M_021_calici_5 (MW684836.1), *Bavovirus* sp. isolate Chicken/NLD/2019/V_M_029_calici_1 (MW684837.1), MAG: *Caliciviridae* sp. isolate CAL/PB31-SI19/Switzerland/2019 (OM469264.1) and Calicivirus chicken/V0021/Bayern/2004 (NC_075411.1). Numbering refers to nucleotides in the indicated genomic sequences.

ORF1* initiates upstream of ORF1 and overlaps it, except in Chicken calicivirus isolate CaliciD62/2013 and *Bavovirus* sp. isolates Chicken/NLD/2019/V_M_021_calici_5, Chicken/NLD/2019/V_M_029_calici_1 and Chicken/NLD/2019/V_M_032_calici_1, in which the ORF1* sequence forms an N-terminal extension of the ORF1 reading frame (continuation indicated by dots). Potential AUG initiation codons within ORF1* are in bold and highlighted blue.

To illustrate sequence similarity, calicivirus ORF1 sequences are aligned below sequences of ORF1* and ORF1*-ORF1 fusion polypeptides, with amino acid residues in these sequences that are identical to the consensus residues in the ORF1*-ORF1 fusions highlighted in yellow.
